# LipidMatch: an automated workflow for rule-based lipid identification using untargeted high-resolution tandem mass spectrometry data

**DOI:** 10.1186/s12859-017-1744-3

**Published:** 2017-07-10

**Authors:** Jeremy P. Koelmel, Nicholas M. Kroeger, Candice Z. Ulmer, John A. Bowden, Rainey E. Patterson, Jason A. Cochran, Christopher W. W. Beecher, Timothy J. Garrett, Richard A. Yost

**Affiliations:** 1 Department of Chemistry, University of Florida, 214 Leigh Hall, Gainesville, Florida 32611 USA; 2 College of Engineering, University of Florida, 412, Newell Dr, Gainesville, FL 32611 USA; 3 National Institute of Standards and Technology, Hollings Marine Laboratory, 331 Ft. Johnson Road, Charleston, SC 29412 USA; 4 Clinical and Translational Science Institute, University of Florida, 2004 Mowry Road, Gainesville, FL 32610 USA; 5 Department of Pathology, Immunology, and Laboratory Medicine, College of Medicine, University of Florida, 1395 Center Dr, Gainesville, FL 32610 USA

**Keywords:** Lipidomics, Data-independent analysis, Data-dependent analysis, Mass spectrometry, High resolution mass spectrometry, Tandem mass spectrometry, Liquid chromatography, Imaging mass spectrometry, In silico libraries, Oxidized lipids

## Abstract

**Background:**

Lipids are ubiquitous and serve numerous biological functions; thus lipids have been shown to have great potential as candidates for elucidating biomarkers and pathway perturbations associated with disease. Methods expanding coverage of the lipidome increase the likelihood of biomarker discovery and could lead to more comprehensive understanding of disease etiology.

**Results:**

We introduce LipidMatch, an R-based tool for lipid identification for liquid chromatography tandem mass spectrometry workflows. LipidMatch currently has over 250,000 lipid species spanning 56 lipid types contained in in silico fragmentation libraries. Unique fragmentation libraries, compared to other open source software, include oxidized lipids, bile acids, sphingosines, and previously uncharacterized adducts, including ammoniated cardiolipins. LipidMatch uses rule-based identification. For each lipid type, the user can select which fragments must be observed for identification. Rule-based identification allows for correct annotation of lipids based on the fragments observed, unlike typical identification based solely on spectral similarity scores, where over-reporting structural details that are not conferred by fragmentation data is common. Another unique feature of LipidMatch is ranking lipid identifications for a given feature by the sum of fragment intensities. For each lipid candidate, the intensities of experimental fragments with exact mass matches to expected in silico fragments are summed. The lipid identifications with the greatest summed intensity using this ranking algorithm were comparable to other lipid identification software annotations, MS-DIAL and Greazy. For example, for features with identifications from all 3 software, 92% of LipidMatch identifications by fatty acyl constituents were corroborated by at least one other software in positive mode and 98% in negative ion mode.

**Conclusions:**

LipidMatch allows users to annotate lipids across a wide range of high resolution tandem mass spectrometry experiments, including imaging experiments, direct infusion experiments, and experiments employing liquid chromatography. LipidMatch leverages the most extensive in silico fragmentation libraries of freely available software. When integrated into a larger lipidomics workflow, LipidMatch may increase the probability of finding lipid-based biomarkers and determining etiology of disease by covering a greater portion of the lipidome and using annotation which does not over-report biologically relevant structural details of identified lipid molecules.

**Electronic supplementary material:**

The online version of this article (doi:10.1186/s12859-017-1744-3) contains supplementary material, which is available to authorized users.

## Background

Lipids are ubiquitous and structurally diverse molecules with numerous biochemical functions. Therefore, the measurement of lipids has diverse applications, especially in the clinical sciences. Most notably, lipids have been shown over the past decade to be valuable as potential biomarkers for several diseases, due to the numerous biological functions of lipids within an organism. This diversity in lipid function is accomplished through diversity in lipid structure [1]. There are over 180,000 possible lipid species, without taking into account all of the possible double bond positions, backbone substitutions, and stereochemistry [2], and several million potential lipids when all these structural differences are accounted for. Thus, one major analytical challenge in lipidomic measurement is the process of identifying lipids across this diverse range of structures and varying abundances, potentially differing up to several million-fold [3].

One of the more promising strategies for comprehensive lipidomics is to utilize ultra-high performance liquid-chromatography with high resolution tandem mass spectrometry (UHPLC-HRMS/MS). UHPLC-HRMS/MS provides molecular specificity using exact mass, MS/MS, and retention time to assign detailed structure to each lipid identification [4]. Obtaining MS/MS spectra provides unique structural information to help identify lipid species that may contain different fatty acid constituents, but the same number of carbons and degrees of unsaturation. These isomeric species often co-elute [5], and therefore are generally indistinguishable by retention time and exact mass alone. MS/MS can provide backbone, fatty acid moiety, and lipid class information, as neutral losses or fragment ions are often produced by cleavage at the linkages between the backbone and fatty acyl constituents of a particular lipid.

In comparison to proteomics, lipidomics is an emerging technique which currently lacks community-wide agreement concerning the best software choice for the comprehensive and accurate identification of lipids based on chromatographic and tandem mass spectrometric data. A major challenge is the limited number of synthesized standards available, making it difficult to cover the much larger variety of lipid structures for MS/MS spectral matching. In the absence of authentic standards, this challenge has been partially ameliorated by developing in silico libraries for acyl-containing lipids. For example, in 2013, Kind et al. released LipidBlast [6], developing a computer generated library of 119,200 lipids across 26 lipid classes, which included predicted mass/intensity pairs.

A second major challenge is the accurate annotation of lipid identifications based on the fragmentation observed [7]. The annotation depends on the structural resolution, which is the structural detail inferred by experimental data, specifically the MS/MS spectra. Structural resolution for lipids is dependent on specific structural characteristics known, such as double bond location, geometric isomerism (*cis* versus *trans*), and the position, lengths and degrees of unsaturation of fatty acyl constituents. For example, if only the exact mass of the precursor and choline head group of a phosphatidylcholine species is observed, the species can only be annotated by total carbons and degrees of unsaturation (e.g. annotated as PC(32:1)) (assuming no overlap from fragmentation of other choline containing species, such as the ^13^C isotopic peaks of SM). If the precursor mass and fatty acyl fragments are observed, then the lipid can be identified by acyl-constituents (eg. PC(16:0_18:1)), with an underscore denoting that the position of the fatty acyl chain on the backbone is unknown. For most lipid types, this is the limit of structural resolution that can be accurately annotated using UHPLC-HRMS/MS without specialized or additional approaches. Currently, most lipidomics software over-report structural resolution, which can lead to incorrect biological interpretation of the data [8].

A third challenge for lipid identification is the fact that features (peaks defined by a mass to charge ratio (*m/z*) and retention time) often contain multiple co-eluting molecules with similar *m/z* values. One common case is lipids sharing the same class, total carbons and degrees of unsaturation, but different acyl constituents, for example PC(18:0_18:1) and PC(16:0_20:1). This overlap reduces spectral similarity scores, which are used for identification by most software.

To overcome these challenges, we have developed LipidMatch. LipidMatch currently contains the most comprehensive lipid fragmentation libraries of freely available software, when ranked by the number of lipid types. LipidMatch includes in silico libraries with over 250,000 lipid species across 56 lipid types, including oxidized lipids. LipidMatch incorporates user-modifiable, rule-based lipid identification, which allows for accurate lipid annotation in regards to structural resolution. In addition, if multiple identifications exist for one feature, LipidMatch outputs include all possible identifications ranked by summed fragment intensities.

## Implementation

LipidMatch was written in R [9]. The user interface for LipidMatch consists of a series of dialogue boxes developed using gWidgets API and the tcltk R package. Users can access LipidMatch as a file in the Additional file 1, with the latest version available at <http://secim.ufl.edu/secim-tools/>. A manual and video tutorials are provided to walk users through the entire lipidomics workflow, including vendor file conversion to open source format, feature processing, LipidMatch identification, in silico lipid library development, and the ability to append identifications from other software (e.g. MS-DIAL or Greazy).

### Generation and validation of LipidMatch in silico libraries

In silico libraries were developed in Excel as described in video tutorial 6 in the Additional file 1. Briefly, an R script was used to generate a list of possible fatty acid combinations for acyl containing lipids with 2 or 3 fatty acids. A list of 39 possible endogenous fatty acids and 214 potential oxidized fatty acids were incorporated (contained in the LipidMatch zip file). Combinations excluded redundant possibilities such as 18:0_20:0 and 20:0_18:0. For oxidized lipids, a list of 126 potential long chain oxidized fatty acids was generated by the addition of one or more (depending on the degrees of unsaturation) O (as a ketone or epoxy), OH (as a hydroxyl radical), and OOH (as a perhydroxyl radical) to unsaturated fatty acids within the list of 39 endogenous fatty acids. A list of 88 potential short chain oxidized fatty acids were generated by cleavage of unsaturated fatty acids contained in LIPID MAPS and addition of a terminal CHO (aldehyde) or COOH (carboxylic acid). Oxidized fatty acyl chains were combined with the original list of fatty acyl chains to generate possible fatty acyl combinations for oxidized lipids.

For each lipid class, structurally indicative fragments were compiled using other MS/MS databases (LIPID MAPS [10], LipidBlast [11], and MS-DIAL [12]), literature, and/or experimentally derived fragmentation. Using multiple sources to obtain fragmentation allowed for cross-validation of fragments and generation of lipid class-specific fragmentation rules (see video tutorial 6 of the Additional file 1 for details). Fragment masses calculated were validated with MS/MS of internal standards obtained using HCD fragmentation [13] on a high-resolution orbitrap mass spectrometer, or literature searches. The following internal standards were used for verification (acronyms are defined in Additional file 2: Table S1): CE(17:0), CE(19:0), CE(2:0), Cer(d18:1/17:0), Cer(d18:1/25:0), MAG(17:0), DAG(14:0/14:0), DAG(19:2/19:2), DAG(20:0/20:0), GlcCer(d18:1/12:0), LPA(17:0), LPC(17:0), LPC(19:0), LPE(14:0), MG(17:0), OxPC(16:0/9:0(CHO)), PA(14:0/14:0), PC(14:1/14:1), PC(17:0/17:0), PC(19:0/19:0), PE(15:0/15:0), PE(17:0/17:0), PG(14:0/14:0), PG(15:0/15:0), PG(17:0/17:0), PI(8:0/8:0), PS(14:0/14:0), PS(17:0/17:0), SM(d18:1/17:0), SM(d18:1/6:0), TAG(13:0/13:0/13:0), TAG(15:0/15:0/15:0), TAG(17:0/17:0/17:0), TAG(17:1/17:1/17:1) and TAG(19:0/19:0/19:0). All internal standards were obtained from Avanti Polar Lipids (Alabaster, Alabama), except TAG species, which were purchased from Sigma-Aldrich (St. Louis, MO), and cholesterol esters, which were obtained from Nu-Chek Prep (Elysian, MN).

### Lipidomics workflow with LipidMatch

**Fig. 1:**
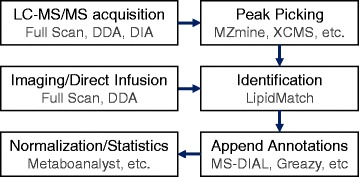
Options for open source software integration with LipidMatch in a lipidomics data processing workflow. Acquisition modes for fragmentation which can be used to annotate lipids with LipidMatch include data-dependent analysis (DDA) and data-independent analysis (DIA) for both direct infusion and liquid chromatography (LC) tandem mass spectrometry (MS/MS) approaches

LipidMatch is designed to be integrated with other open-source software to streamline the lipidomics workflow as described in Fig. 1. LipidMatch was developed and tested using data acquired from a Q-Exactive orbitrap mass spectrometer (Thermo Scientific, San Jose, CA). LipidMatch has also been tested using data acquired on an Agilent 6540 Q-TOF (Agilent Technologies, Santa Clara, CA). LipidMatch can be used with a variety of other vendors and data formats. Ion selection techniques used to acquire fragmentation, including all-ion-fragmentation (AIF), inclusion list-based targeted approaches, and data-dependent topN (ddMS^2^-topN) approaches can be used with LipidMatch to annotate lipids acquired using liquid chromatography, direct injection, or imaging approaches. LipidMatch is not recommended for most applications using low resolution mass spectrometers. For brevity, we will focus on UHPLC MS/MS methods using the data-dependent topN approach, although video tutorials for imaging approaches and AIF approaches are included in the Additional file 1.

In the workflow recommended for LipidMatch, users acquire full scan data for all the samples in negative and/or positive polarity. In addition, users acquire ddMS^2^-topN spectra from pooled samples or from other representative samples. Using iterative exclusion (IE) [13] on the pooled or representative samples can increase the number of ions with respective fragmentation spectra. This is highly recommended if spectra are dense (many overlapping lipid signals).

Following data-acquisition, the full scan data (either centroid or profile) can be processed to determine features, defined as an ion or ions sharing the same *m/z* and retention time. Features can be determined from various peak picking software such as MZmine [14], XCMS [15], or MS-DIAL [12]. The feature table can have nearly any format, allowing flexibility in choosing feature processing workflows. Video tutorial 2 explains how users can process data using MSConvert [16] and MZmine 2.20 using a batch file for MZmine. The batch file was optimized for lipids using the chromatographic methods in Additional file 2: Table S2 and is included with the tutorial videos in the LipidMatch file.

**Fig. 2:**
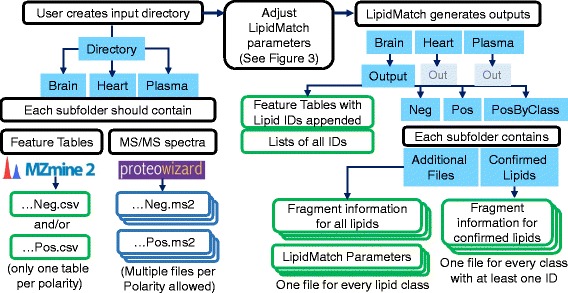
Workflow for using LipidMatch, with input and output folder structure and files. *Green* boxes represent .csv files, *dark blue* boxes represent open source MS^2^ files (.ms2), and filled *light blue* boxes represent folders. Three stacked boxes represent that multiple files are allowed or generated. The subfolders (brain, heart, and plasma) are examples, these folders can be for any biological substrate. In addition if only one biological substrate is analyzed, only the main directory folder is needed. In the outputs generated by LipidMatch each subfolder contains an output folder as depicted above

Once feature tables are created for each biological substrate and each polarity, features can be directly annotated using LipidMatch and the previously generated MS/MS data. Peak picking of MS/MS data and conversion to .ms2 file format should be done using MSConvert [16]. Feature table(s) and MS/MS data are placed into a directory as shown in Fig. 2. Often researchers may have multiple feature tables, one for each polarity type and feature tables for each substrate analyzed. Users can include a subfolder for each sample type, and feature tables should end in “n.csv” or “neg.csv” (not case sensitive) for negative mode, and “p.csv” or “pos.csv” for positive mode. Each folder should contain respective MS/MS data for that substrate in .ms2 format also ending in “neg.csv” or “pos.csv”, depending on polarity. The file should have “dd” in the name if it is data-dependent (DDA) data or targeted data, and “AIF” if it is all-ion-fragmentation data. For example, the user could create a folder for a lipidomics experiment on cancer, with two sub-folders, one for plasma from cancer patients and non-cancer patients and one for healthy tissue and tumor tissue. Each sub-folder could contain, for example, 2 DDA .ms2 files in positive mode and 2 DDA files in negative mode, one pooled for participants with cancer and one pooled for non-cancer participants, as well as the corresponding feature tables in negative and positive polarity. Once the user runs LipidMatch and enters user parameters, LipidMatch will automatically append identifications to each feature table using MS/MS files contained in that feature table’s subfolder.

Once lipid identifications are obtained using LipidMatch, identifications from any other software such as Greazy [17], LipidSearch (Thermo Scientific, San Jose, CA), and MS-DIAL can be appended in additional columns to the feature table (Fig. 1). The annotations are appended from one file to another if the retention time and *m/z* of a feature in one table matches the retention time and *m/z* of a feature from a second table within a user defined mass tolerance and retention time tolerance. For example, if a retention time tolerance of 0.1 min and mass tolerance of 10 ppm is used, a feature annotated PE(36:2) + H with a retention time of 6.72 and *m/z* of 744.5536 will be appended to a feature generated by a different software with a retention time of 6.68 and *m/z* of 744.5540. Lipidome coverage and confidence in identifications can be increased by appending identifications from multiple software onto one feature table. In addition, metabolite, xenobiotic, or other identifications from software such as Compound Discoverer (Thermo Scientific, San Jose, CA) or MS-DIAL can be appended for a more global approach. Furthermore, lipidome coverage can be increased by the user community by adding new in silico fragmentation libraries. Libraries for LipidMatch can be developed using LipidBlast Templates [11] or as explained in video tutorial 6 found in the Additional file 1. Each library should be developed with the correct annotation based on the structural resolution that can be inferred by fragments chosen for the identification criteria.

### LipidMatch inputs and operations

**Fig. 3:**
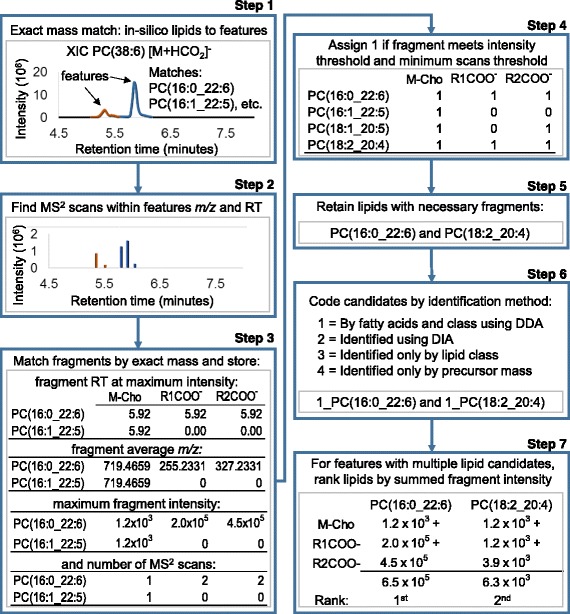
Simplified flow diagram of LipidMatch operations. The steps for identification of the feature at *m/z* 850.5604 and retention time (RT) 5.92 as formate adducts of PC(16:0_22:6) and PC(18:2_20:4) are shown as an example in *grey* boxes for each step. Note that the number of lipid identifications and fragments queried in the example are reduced significantly for illustration purposes. For Step 5, R1COO^−^ and R2COO^−^ were required for identification above an intensity threshold of 1000 in at least one scan across the peak

LipidMatch user inputs and respective operations are exemplified in Fig. 3 using experimental data for PC(38:6) [M + HCO_2_]^−^. A similar schematic to Fig. 3, which includes user inputs and modifiable parameters, is provided in the Additional file 1 (Additional file 2: Fig. S1). The user first chooses directories containing feature table(s), for example those generated by MZmine (Fig. 2). Then, LipidMatch performs exact mass matching at the MS1 level between in silico precursor ions and each features *m/z* using a user defined *m/z* tolerance (Da) (Step 1; Fig. 3). Precursor ions include all adducts contained in the in silico libraries for the respective polarity, but do not include dimers, multimers or in-source fragments. Each feature and lipid match will be termed a “feature-lipid pair”. MS/MS scans from .ms2 files within a user defined retention time and *m/z* tolerance of each feature is determined (Step 2; Fig. 3). The *m/z* tolerance is the same as the isolation window used for selecting ions.

For each MS/MS scan of each feature, experimental fragments are matched against in silico lipid fragments *m/z* using a tolerance window (ppm). The total number of scans across a feature containing that fragment is calculated. In addition, the fragments average *m/z*, maximum intensity, and retention time at maximum intensity across all scans are calculated for a feature (Step 3; Fig. 3). This information on fragments for each feature-lipid pair is saved as a table in .csv format for each lipid class. Each fragment is assigned 1 if it is above the user defined minimum intensity and scans threshold and 0 if the fragment does not meet these criteria or was not found within the *m/z* tolerance (Step 4; Fig. 3). The default number of scans required is 1 based on orbitrap mass spectrometers, but can be increased for other applications. The user modifiable intensity threshold for fragment ions to be considered real is dependent on the mass analyzer, the type of detector and the noise level.

In Step 4, fragments assigned a 1 are considered observed based on the threshold criteria discussed above. Lipids are identified if they contain the necessary observed fragments. For example, for PCs measured as formate adducts, both negative ions of the fatty acyl constituents must be observed (Step 5 of Fig. 3), while for protonated PCs the PC head group ion 184.0733 must be observed, along with at least one fatty acyl indicative fragment if the lipid is to be characterized at the level of fatty acyl constituents. Default fragments which must be observed for each lipid class were determined using high collisional induced dissociation (HCD) on a Q-Exactive orbitrap mass spectrometer of internal standards, or endogenous lipids verified in literature. Users can modify which fragment ions for each lipid class must be observed for identification using a simple Excel sheet as outlined in the 6th video tutorial. In certain cases it may be important to optimize fragment criteria for applications not employing HCD fragmentation with an orbitrap analyzer. Experimental protocols including mobile phase (adducts observed), low and high mass cutoff, resolution, and type of fragmentation (e.g. HCD, CID, or UV) will determine what fragment ions are necessary for each lipid type to be identified. Therefore, for applications other than those using HCD fragmentation and orbitrap detection, we strongly recommend checking the existing fragmentation rules against MS/MS obtained in-house. Fragments chosen for confirmation should be of relative high intensity and distinguish the lipid structure from other lipids with similar fragmentation. It is important to note that while fragmentation measured on other high resolution instruments, such as qTOF platforms, can result in significant changes in the relative fragment intensities, in most cases the fragment masses observed are the same. Therefore, since LipidMatch does not include intensity in in silico fragmentation libraries and does not include relative intensities in identification, criteria for identification will often be similar between instruments.

After lipids are identified, they are assigned a number based on whether they are identified by class and fatty acyl constituents (1), by data-independent analysis (2), only by class (3), or only by precursor *m/z* without fragment matching (4) (Step 6, Fig. 3). If multiple lipids are identified for a single feature, the lipids are ranked by the summed intensity of all their fragments with in silico fragment exact mass matches, including those not used for confirmation (Step 7, Fig. 3). The final ranked lipid identifications are appended onto the feature table, along with the lipid class and adduct of the top ranked lipid and summed fragment intensities for each identification.

## Results and discussion

### Comparison of lipid software features

Table 1 compares features in LipidMatch, MS-DIAL, Greazy, and LipidSearch which can all be used to analyze UHPLC-HRMS/MS data (note that this is not a complete list of available lipidomics software). LipidMatch, MS-DIAL, and Greazy are open source, while a license must be purchased for LipidSearch.

**Table 1:** Comparison of lipid identification software

	LipidMatch	MS-DIAL	GREAZY	LipidSearch 4.1
Identification (ID) Strategy*	Rules	Similarity	Similarity	Rules & Similarity
Fragment Intensity for ID*	Yes (ranking)	Yes	No	Yes
in-silico Library (Types)	56	34	24	59
User Developed Libraries	Yes	Difficult	Difficult	Difficult
Programming Language	R	C#	C++	Java
Restrictions	None	None	None	Purchase License
Multiple Vendor Formats	Yes (.ms2)	Yes (.abf)	Yes (.mzML)	Yes (vendor DLL)
Data Independent Analysis**	Yes	Yes	No	No
MS^3^ analysis	No	No	No	Yes
Multiple Hits in Final Report	Yes (ranked)	No	No	Yes (ranked)
Structural Resolution***	Correct	Over Reports	Over Reports	Correct
Identifiers (eg. LipidMaps)	No	Yes	No	No
Computational time (HR data)	Slow	Medium	Fast	Fast
Employs False Discovery	No	No	Yes	No

Note that in determining total types of lipids contained in each software’s in silico library all ether linked lipids contained were considered two types (plasmenyl and plasmanyl) and all oxidized lipids contained across numerous classes were considered one lipid type

*Please read text for further information

**Not discussed in-depth in this manuscript. LipidMatch can be applied to AIF data independent analysis (currently only supports Thermo files), while MS-DIAL can be applied to AIF and SWATH approaches

***Correct reporting of structural resolution means that lipids are annotated only at the level of structure known based on fragmentation

Currently, MS-DIAL and LipidSearch provide the most user-friendly interfaces and ease of use. In contrast to other UHPLC-HRMS/MS identification software, LipidMatch is completely written in R. Compared to the other lipid identification software written in middle level languages, such as C++, LipidMatch can take longer to run, especially for high resolution data. This is due to the slow speed of imbedded for-loops in R and the extensive LipidMatch libraries and hence large search space. While run time can be longer, LipidMatch can readily be integrated with diverse R tools and statistical packages available for mass spectrometry and omics-based studies.

Databases for lipid identification differ both in coverage and information type. For example, LipidMatch and Greazy databases contain only the exact *m/z* of precursor ions and fragment ions, while MS-DIAL and LipidSearch include simulated intensities. In addition, software such as MS-DIAL and LipidMatch contain static in silico libraries, while libraries in Greazy are generated as the program is executed, based on the types of lipids and fatty acyl chains the user specifies. While LipidMatch libraries are static excel files, as with all four software previously mentioned, the user can select which lipid types to query using LipidMatch, hence limiting searches only to biologically relevant or expected lipid types and reducing run time. LipidMatch libraries contain only exact *m/z* values of precursors and fragment ions, making it relatively trivial for users to generate in silico libraries and/or convert other databases to the LipidMatch library format. LipidMatch contains all lipid types in MS-DIAL 2.24, as well as LipidBlast release 3 development libraries. With 56 lipid types, LipidMatch in silico libraries cover the greatest number of lipid types of any open source software to date, with MS-DIAL containing 34 lipid types, and Greazy containing 24 lipid types (Table 1).

All four programs use different identification strategies. MS-DIAL and LipidSearch include intensity to rank lipid identification by a similarity score. Greazy includes a similarity score as well as a false discovery probability based on the total number of fragments observed, thus solely relying on *m/z*. Both LipidMatch and LipidSearch include rule-based identification, which allows correct annotation of lipid structure based on fragments observed (correct structural resolution). While all other open-source software sort identifications by similarity score, LipidMatch sorts lipid identifications by summed fragment intensity. For each lipid species identified, all expected fragment ions are summed (using the scan with the highest intensity for each fragment). Fragment ions to sum are determined from the in silico fragment *m/z* values for that species and include fragments not necessary for lipid identification (for example the loss of the PC head group for PCs when the *m/z* 184.0733 PC fragment is observed). For each feature, the lipid ions are ranked from maximum to minimum summed intensity.

LipidMatch ranking is based on the assumption that a feature often represents multiple lipid ions and that ranking is meant to determine the relative signal contribution of each lipid to the feature. In other software, by using similarity score, ranking is based on which lipid identification is most confident. While both ranking algorithms produce similar results in many cases (see section below: *A case study: Identification of lipids in red cross plasma*), LipidMatch algorithm is designed based on a more accurate assumption of multiple co-eluting lipids sharing *m/z* values within the same accurate mass. In simple dot product matching, the algorithm is based on the assumption that the fragmentation spectra is solely based on the ion of interest. Any deviation from the predicted fragmentation spectra, such as additional high intensity fragment peaks from co-eluting isobaric species, will reduce the dot product score. Many lipids will not be identified due to co-eluting isobaric species adding more fragments to the spectra and hence reducing the dot product score. MS-DIAL has approached this issue by reducing the impact of peaks not contained in the in silico fragmentation library on the modified dot-product score. Fragments from different species which overlap in exact mass, for example fatty acyl fragments from 18:0 in TG(18:0/18:0/18:0) and TG(16:0/18:0/20:0), will still decrease the modified dot-product score in MS-DIAL, and hence lead to false negatives.

**Fig. 4:**
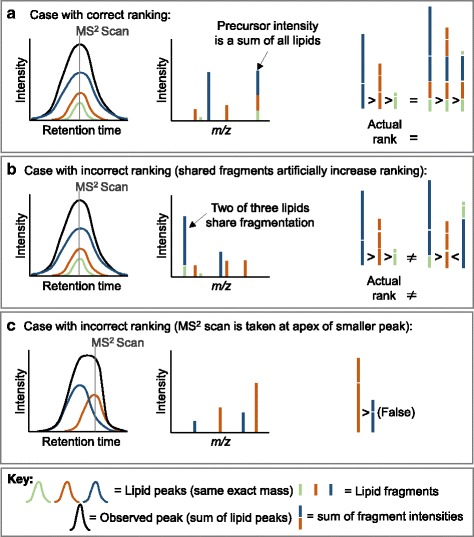
Problematic cases which can arise when ranking lipids by the sum of fragment intensities. The first panel (**a**) represents a case were lipids are accurately ranked (*far right*) based on the areas under the peak (*far left*). It also show that even in this case, the precursor intensity doesn’t reflect a single intensity, but a sum of the intensity of all precursor isomers (*middle*). In panel (**b**) two lipids (*blue and light gree*n) share a high intensity fragment with the same *m/z* (*middle*), inflating their intensity values leading to false ranking (*far right*). In panel (**c**) the MS/MS scan misses the apex of the lipid with a blue trace, and hence the summed intensity for the blue trace is reduced

Ranking lipid identifications for a given feature is complicated by overlapping mass spectral fragments in LipidMatch as well. A number of problematic cases can arise. For example, for a given lipid type with high intensity fragments below the *m/z* cutoff, the ions summed fragment intensity will be reduced compared to lipid species with the bulk intensity of fragments within the *m/z* range. Similarly, if high intensity fragments are missing from the in silico library for a lipid type, these lipids will be artificially lowered in their ranking in terms of contribution to feature signal. In addition, shared fragment ions for some lipids will artificially inflate summed fragment intensity (Fig. 4b) and fragment intensity will depend on the MS/MS scans proximity to a given ions apex (Fig. 4c). Similarity score matching, such as that used by MS-DIAL, suffers similar problems.

To determine the accuracy of lipid rankings and identifications using LipidMatch, identification of lipids in Red Cross plasma using LipidMatch was compared to MS-DIAL and Greazy. Lipid software excluded for comparison included LipidSearch (Thermo Scientific), Lipidyzer (SCIEX), and SimLipid (PREMIER Biosoft), which are not open source software, and Alex [18], LipidXplorer [19], MS-LAMP [20], LIMSA [21], LOBSTAHS [22], Lipid Data Analyzer [23], LipidQA [24], and Lipid-Pro [25], which were not designed for UHPLC-HRMS/MS untargeted experiments. As stated previously, LipidMatch, MS-DIAL, and Greazy differ in lipid identification strategy; hence, the amount of features with the same identifications between LipidMatch and the other software platforms was used to assess the accuracy of the LipidMatch ranking algorithm. Further work, with spiked co-eluting standards sharing the same exact mass at varying concentrations would be helpful to further assess the ranking algorithm accuracy.

### A case study: Identification of lipids in red cross plasma

LipidMatch, Greazy, and MS-DIAL were applied to five replicate injections of Red Cross blood plasma. Data was acquired in positive and negative polarity, using iterative exclusion [13] and data-dependent top 5 (ddMS^2^-top5) to acquire MS/MS fragmentation. Liquid chromatography and mass spectrometer parameters are shown in Additional file 2: Tables S2 and Table S3, respectively. Identifications from all software were appended to the MZmine feature table using the CombineSoftwareIDs.R script. Both the script, MZmine parameters (batch file), and an excel sheet with the resulting annotations of features across all 3 software (Additional file 3: Table S4) are included in the Additional file 1. The script aligns features with similar *m/z* (10 ppm window used) and retention times (0.2 min window used) from two different peak picking or identification software.

Compared to the other major open-source software platforms, such as MS-DIAL and Greazy, LipidMatch annotated many more lipid ions. LipidMatch was used to identify 210 lipid ions across 159 features and 15 lipid types in negative polarity. In positive ion mode, LipidMatch was used to annotate 5159 unique lipid ions across 1401 features and 26 lipid types. The large number of unique lipid ions in comparison to a smaller amount of identified features is due to overlap of co-eluting lipids sharing the same exact mass, allowing for multiple lipids identified for a given feature. It is important to note that annotations of class-specific fragments (as indicated by “3_” in Additional file 3: Table S4), are significantly more tentative than identifications using fatty acyl fragments. This is especially true for choline containing lipid classes such as SM and PC, which share common fragments. For positive ion mode, 987 features were annotated with fatty acyl information. It is also important to note that in this study, we look at the number of lipid ions annotated, including multiple adducts for a given lipid species. When only unique lipid molecules were taken into account by manually removing redundant adducts and features, and identifications using only choline specific fragmentation were removed, a total of 728 features with unique lipid molecular annotations were identified by LipidMatch for this dataset, as has been published previously [13]. The curated 728 lipid molecular identifications using LipidMatch is still significantly greater than the total lipid ions identified by MS-DIAL and Greazy combined. Additional file 3: Table S4 includes all features identified in Red Cross plasma, with LipidMatch, MS-DIAL, and Greazy annotations.

MS-DIAL and Greazy identified 143 and 94 features in negative mode, respectively, and 411 and 180 features in positive mode, respectively. Lipid types identified, which were unique to LipidMatch, included oxidized species (151 across TG, PC, and LPC in positive polarity), plasmenyl and plasmanyl TGs (19 species in positive mode), sphingosines (2), sulfatides (1), and PI species in positive mode as ammonium adducts (18). It is important to note that many additional unique in silico libraries exist in LipidMatch, for example cardiolipin as ammonium adducts, but these species are not observed in plasma samples. Bar graphs displaying the number of lipid species in each lipid type identified by LipidMatch, MS-DIAL, and Greazy, and overlapping identifications between software are shown in Additional file 2: Fig. S2 (negative polarity) and Additional file 2: Fig. S3 (positive polarity). In addition, pie charts showing the lipid types covered by LipidMatch are shown in Additional file 2: Fig. S4 (negative polarity) and Additional file 2: Fig. S5 (positive polarity).

Since Greazy is limited to glycerophospholipid species, only 65 features in negative polarity and 68 features in positive polarity had identifications across all software. In negative polarity, 97% of these features had the same identification at the structural resolution of fatty acyl constituents across all 3 software platforms. In positive polarity, 71% of features with identifications across all software tested were the same. Note that plasmenyl and plasmanyl species with differences in one degree of unsaturation were considered the same identification due to minimal difference in MS/MS spectra. The greater discrepancy in identifications in positive mode is most likely to do to the low abundance of acyl chain fragments for glycerophospholipids in positive mode, thus making identification by fatty acyl constituents difficult. At the structural resolution of lipid class and total carbons and double bonds, 94% of features contained the same identifications across all 3 software platforms in positive polarity, and 100% of features were identified the same in negative polarity.

Of all lipid types identified by both MS-DIAL and LipidMatch, TGs had the most discrepancy. Of the 136 features identified as TGs by both LipidMatch and MS-DIAL (both sodiated and ammoniated forms), 100% of the top hits were the same at the structural resolution of total carbons and degrees of unsaturation, but only 61% of the top hits were the same at the structural resolution of fatty acyl constituents. TG identification is complicated by the number of co-eluting isomers, for example, LipidMatch identified over 20 co-eluting TG isomers for a number of features. These co-eluting isomers can share one or more fatty acyl constituents, and therefore share common fragments, further complicating identification.

LipidMatch had a significant number of lipid identifications by fatty acyl constituents corroborated by at least one other software, suggesting that LipidMatch identification and the ranking strategy results in similar identifications for glycerophospholipid species compared to other identification algorithms. For the 68 features identified by all software in positive polarity, 92% of identifications by LipidMatch were corroborated by at least one other software. MS-DIAL and Greazy had 86% and 84% of identifications corroborated for these features by at least one other software, respectively. In negative polarity, 98% of LipidMatch identifications (all except one) were corroborated by at least one other software, with MS-DIAL having 98% identifications corroborated and Greazy having 100% of identifications corroborated.

## Conclusion

LipidMatch is a freely available tool with the potential to be incorporated into a diverse range of lipidomics workflows, including imaging, direct-infusion, and LC-MS/MS experiments with both low and high mass resolution. For LC-MS/MS workflows, LipidMatch can be used with any feature processing software, such as MZmine, XCMS, or MS-DIAL. LipidMatch contains the greatest diversity in lipid types of any current open-source software platform and a unique rule-based strategy for identification and summed fragment intensity based strategy for ranking top hits. Compared to other software, LipidMatch is highly customizable. For example, users can select which fragments are necessary for confirmation and develop their own fragmentation libraries in Excel. Additional tools allow the user to pool results from multiple identification software platforms into one feature table. Compared to MS-DIAL and Greazy, LipidMatch was found to provide the most lipid identifications for Red Cross plasma. For features with identifications using all 3 software platforms, identifications were comparable at the level of fatty acid constituents. 92% and 98% of LipidMatch identifications were corroborated by at least one of the other software platforms in positive and negative mode, respectively.

### Availability and requirements

**Project name:** LipidMatch

**Project home page:** http://secim.ufl.edu/secim-tools/

**Operating system(s):** Windows (tested on Windows 7 through 10)

**Programming language:** R

**Other requirements:**

1) R version: 3.3.3

2) MSConvert (or other file conversion software capable of generating .ms2 files):

3) MZmine, XCMS, MS-DIAL or other peak picking software

**License:**

4) License: Creative Commons Attribution 4.0 International (CC BY 4.0) https://creativecommons.org/licenses/by/4.0/.

## Additional files

Additional file 1: LipidMatch Software. The 2017_6_14_LipidMatch_Distribution.zip file contains lipid libraries in .csv format, a batch file for lipidomics with MZmine processing, the LipidMatch R script, and additional helpful R scripts for lipidomics data processing. The .zip file also contains files to guide the user in using LipidMatch, which include: video tutorials, a manual, a trouble shooting document, and example input and output data. For the most up to date version of LipidMatch please visit: http://secim.ufl.edu/secim-tools/. (ZIP 376634 kb)

Additional file 2: Supplemental Figures and Tables. Contains **Figure S1** through **Figure S5**, and **Table S1** through **Table S3**. (PPTX 593 kb)

Additional file 3: Table S4. Lipid Annotations for Red Cross Plasma Using Three Open Source Software. This excel workbook contains a worksheet for lipid annotations in negative polarity and a worksheet for lipid annotations in positive polarity. Lipids were annotated using LipidMatch, MS-DIAL, and Greazy. For comparison, the resulting identifications were aligned to features determined using MZmine. Data was acquired using iterative exclusion data-dependent top5 (IE-ddMS2-top5) analysis of 6 injections of Red Cross blood plasma. (XLSX 977 kb)

## Abbreviations

AIF: All ion fragmentation
CID: Collision induced dissociation
Da: Dalton
DDA: Data-dependent analysis
ddMS2-topN: Data-dependent top N
HCD: Higher-energy collision induced dissociation
IE: Iterative exclusion
LC-MS/MS: Liquid chromatography tandem mass spectrometry
LPC: Lysophosphatidylcholine
*m/z*: mass to charge ratio
MS/MS: Tandem mass spectrometry
PC: Phosphatidylcholine
PI: Phosphatidylinositol
ppm: Parts per million
TG: Triacylglyceride
UHPLC: Ultra-high-performance liquid chromatography
UHPLC-HRMS/MS: Ultra-high-performance liquid chromatography high resolution tandem mass spectrometry
UV: Ultraviolet
