# Supplementary material for: LipidMatch: an automated workflow for rule-based lipid identification using untargeted high-resolution tandem mass spectrometry data
Source: BMC Bioinformatics. 2017 Jul 10;18:331. doi: 10.1186/s12859-017-1744-3 (PMC5504796; doi:10.1186/s12859-017-1744-3)
Supplement: Supplementary file 1 — LipidMatch Software. The 2017_6_14_LipidMatch_Distribution.zip file contains lipid libraries in .csv format, a batch file for lipidomics with MZmine processing, the LipidMatch R script, and additional helpful R scripts for lipidomics data processing. The .zip file also contains files to guide the user in using LipidMatch, which include: video tutorials, a manual, a trouble shooting document, and example input and output data. For the most up to date version of LipidMatch please visit: http://secim.ufl.edu/secim-tools/. (ZIP 376634 kb) [file 12859_2017_1744_MOESM1_ESM.zip › 2017_6_14_LipidMatch_Distribution/2017_6_15_LipidMatch_Manual.docx]

LipidMatch Processing Manual

Jeremy P. Koelmel, Nicholas M. Kroeger, and Candice Z. Ulmer

Step 1: Install Necessary Programs

- R, version 2.0.3 to 3.3.3 (**3.3.3 is suggested**, do not download the latest version of R as suggested in the tutorial video; this will cause errors) < <http://cran.rstudio.com/>>
- MSConvert (ProteoWizard) <<http://proteowizard.sourceforge.net/downloads.shtml>>
- MZmine, XCMS, MS-DIAL or other peak picking software
- Unzip LipidMatch folder (right click "extract all" in windows 10) and delete zipped file

Step 2: File Conversion using MSconvert

*Note: Depending on the vendor format, file conversion parameters and methods differ. Note that LipidMatch has only been tested using Thermo .raw files and Agilent .d files. Currently LipidMatch does not work for Waters .raw files. An alternative method for file conversion can be found in the troubleshooting document. This alternative is necessary for certain Agilent .d files to prevent an error.*

File Conversion in MSConvert (Thermo Parameters)

- MS2 for R script
  - Upload .raw
  - Peak Picking: Level: 2-2
  - threshold: absolute .0001 most intense
  - msLevel: 2-2
  - Output: MS2
  - *Put all MS2 files in one folder along with the inclusion lists*
- mzXML for mzMINE or XCMS *[or you can use any other alignment software]*
  - Upload .raw
  - Peak Picking: Level 1-1
  - msLevel: 1-1
  - threshold: absolute .0001 most intense
  - Output: mzXML


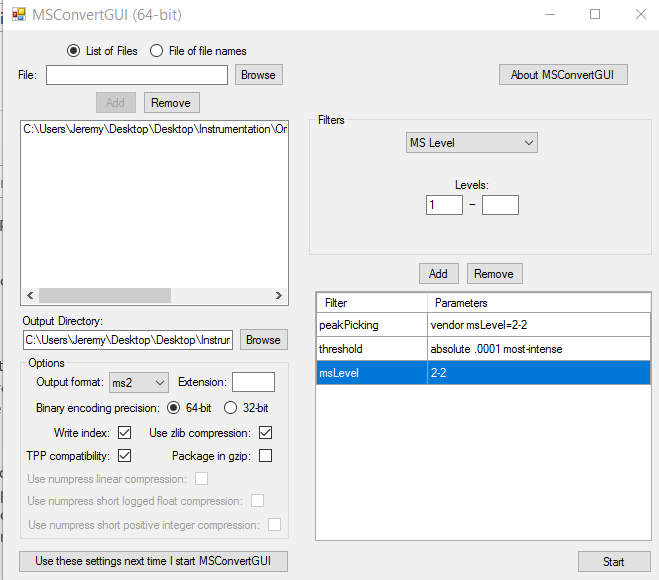
Thermo Parameters for MSconvert:

File Conversion in MSConvert (Agilent Parameters)

- MS2 for R script
  - Upload .d
  - Select 32 bit conversion, and deselect zlib compression
  - Peak Picking: Level: 2-2
  - Output: MS2
  - *Put all MS2 files in one folder along with the inclusion lists*
- mzXML for MZmine or XCMS *[or you can use any other alignment software]*
  - Upload .d
  - Select 32 bit conversion, and deselect zlib compression
  - Peak Picking: Level 1-1
  - Output: mzXML

Agilent Parameters for MSConvert:
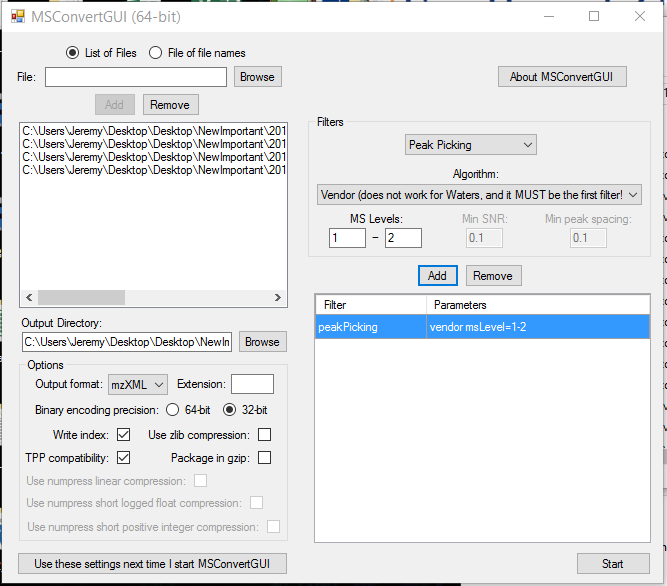


Step 3: Create Feature Table (see example in "ExampleData" subfolder)

- MZmine 2.23 (alternative feature processing software can be used)
  - import mzXML files: raw data methods -> raw data import
  - process files: highlight files, select project -> batch mode -> load batch file (w/ processing parameters). Note the batch file is the .xml file in the LipidMatch folder.
  - A popup box may appear, select "replace"
  - In the "please set the parameters" window, two options for export data as csv exist. Select each one and click Configure on the right side of the popup box.
    - select the following below (you can choose to set one as peak height and the other as peak area)


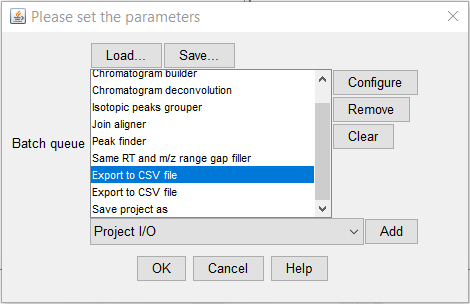


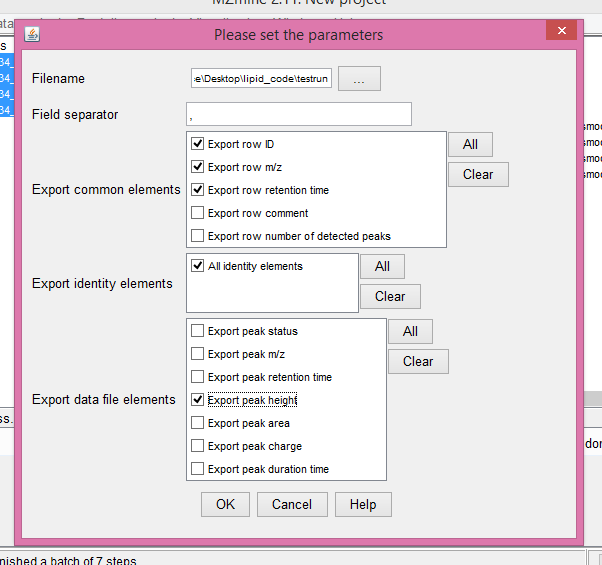


Step 4: Organize data

- Place the .ms2 files containing the MS/MS data in an input folder (only a few representative MS/MS files are needed for each polarity) and the table of features, peak areas, etc. (you may have positive or negative mode data, or both in this folder).
  - One feature table should be included for each polarity run (and no more than one). All files (.ms2 and .csv) should end in N (for negative mode) or P (for positive mode).
  - If the files are acquired using MS^E^ or AIF, have AIF in the file name.
  - If data was acquired using data-dependent analysis or using a targeted list, put dd somewhere in the filename. (e.g. for one folder: featureTableP.csv, featureTableN.csv, 1_ddN.ms2, 2_ddN.ms2, 1_ddP.ms2, 2_ddP.ms2...).
  - **Note that .ms2 file names should not be over 23 characters long**.
- Note that you can include multiple folders in the input directory (see figure below), each with their own feature table (Negative and/or Positive) and MS/MS files. All folders will automatically be included for analysis by LipidMatch.


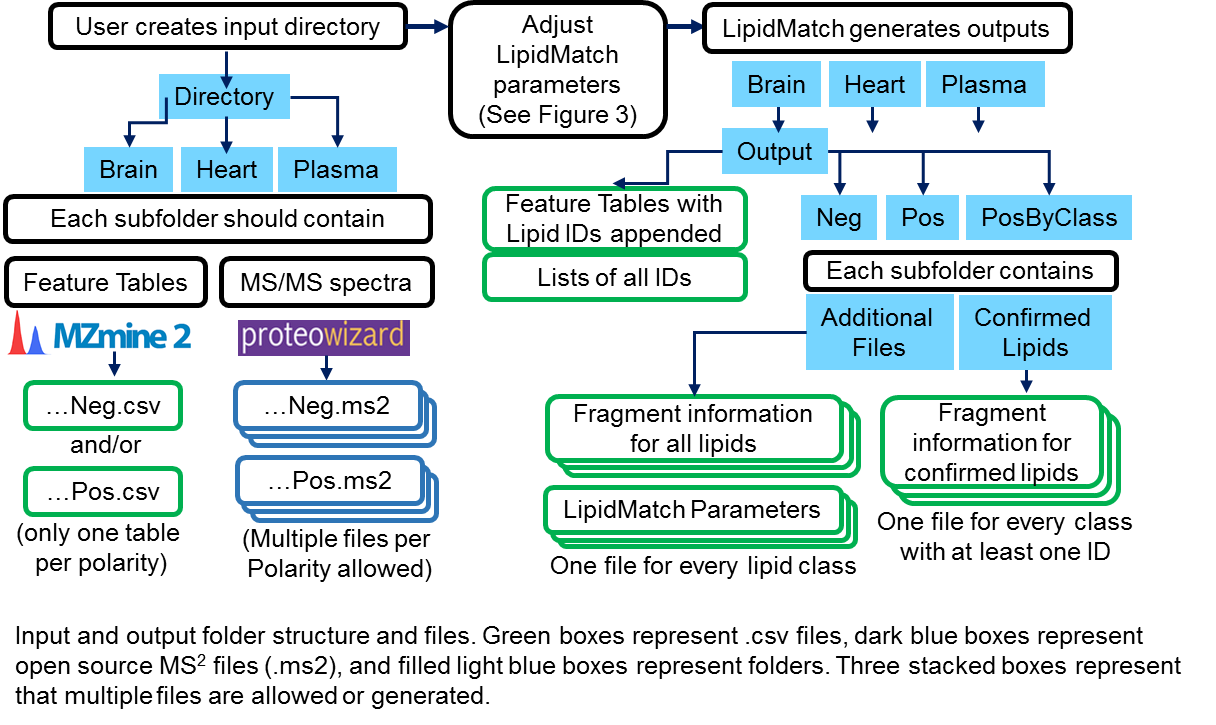


- Open the code ([date]_LipidMatchEnhanced.R), read the notes at the top of the script, select: Ctrl+Shift+S (or Ctrl+A and run)
- A number of pop-up boxes will appear, read the dialogue box and enter the correct information. You may select ‘d’ for most parameters which will use defaults based on an orbitrap experiment with ddMS2 at 35,000 resolution and UHPLC reverse phase chromatography. After the final pop-up box, the code will begin to output results in a folder named “output” which automatically populates in the input folder.

*Note: The pop-up boxes may appear behind all open windows, so minimize all open windows if pop-up boxes are not visible. The image below shows input parameters and how they relate to LipidMatch processes. Note for Agilent files to set minimum intensity to "1".*


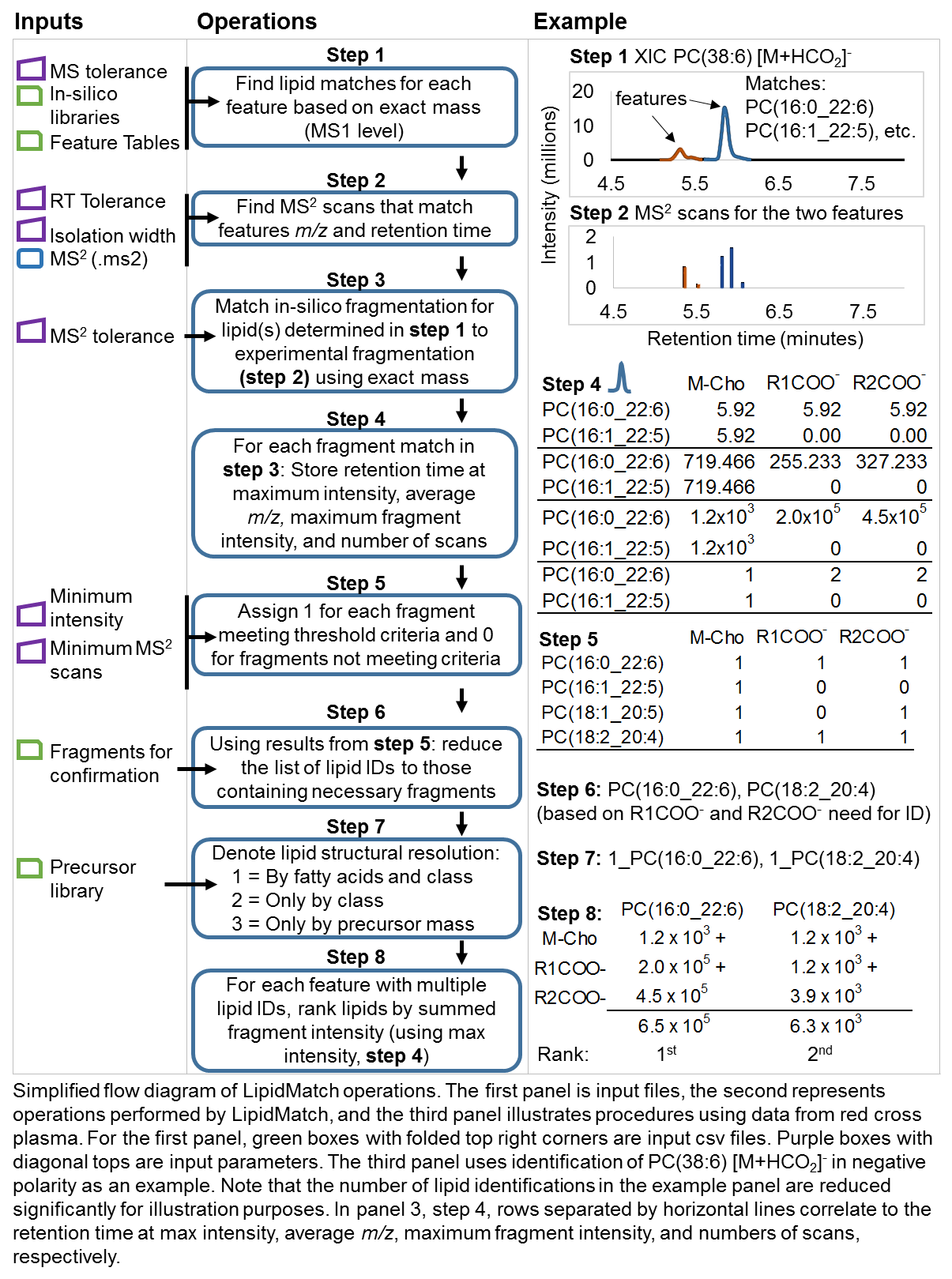


Step 5: Interpreting the results

- Results after Step 4 will be saved in a .csv format which can be opened with excel. The final data is in a filename called either NegIDed or PosIDed. Appended in the next column after the original data from the feature table is a column labeled ID. IDs are contained in this column, and encase of multiple IDs are separated by “I”. In addition “NegIDed”, and “PosIDed”, contain all identification without alignment to the original data.
- There are currently 4 identity markers:
  - 1_[ID] represents confirmation by ddMS2
  - 2_[ID] represents confirmation by AIF
  - 3_[ID] represent ddMS2 by clas
  - 4_[ID] represents confirmation by exact mass.
- The order for multiple confirmations is based on summed fragment intensity.
- The data files in the folder “…Neg/Confirmed/”, “…Pos/Confirmed/”, and “…PosByClass/Confirmed/” can be used to better determine confidence and the most abundant lipids defining a specific feature (these tables contain fragment intensities, retention times at max intensity, experimental and actual mass of fragments, number of scans containing fragments, etc.).

Developing your own in-silico MS/MS libraries or choosing which adducts/ions to search against

- Copy the "LipidMatch_Libraries" subfolder in LipidMatch, and rename this folder (so as not to write over the original). In addition to these instructions video tutorial 6 covers adding new libraries in more detail.
- To change which ions are queried: Find and open the .csv file titled: LIPID_ID_CRITERIA.csv in your new subfolder with all the LipidMatch libraries. Under column D titled "Run DDA" change values to TRUE for lipids you want to search against or FALSE for lipids you want to remove from being searched against. The same applies to column E labeled "run AIF".
- To change which fragments are necessary for confirmation, add fragments by the columns associated with them in their respective .csv file. For example if you double click "LPC_H.csv", column 2 refers to the precursor [M+H]+ ion, column 3 refers to MH-PC, column 4 refers to MH-Tail1+H2O, etc. In the LIPID_ID_CRITERIA.csv column 2 is used to list all fragments (by column number as noted above) which are necessary for confirmation, and column 3 is used to list fragments, with at least one fragmented needed to be observed for confirmation.
- For developing your own MS/MS libraries to search against: Please see the format of the MS/MS libraries by selecting any .csv in the "libraries". You can generate your library accordingly using the video tutorial for more in-depth instructions. The first column should be the name of the compound with the header the class acronym, the second column the precursor mass, and any number of columns thereafter will be fragment ions (even if precursors do not show up in MS/MS they are needed for exact mass matching and determining MS/MS scans in .ms2 files). Put all lipid libraries into one folder and add a row to LIPID_ID_CRITERIA.csv with information for any new libraries.
