# Supplementary material for: LipidMatch: an automated workflow for rule-based lipid identification using untargeted high-resolution tandem mass spectrometry data
Source: BMC Bioinformatics. 2017 Jul 10;18:331. doi: 10.1186/s12859-017-1744-3 (PMC5504796; doi:10.1186/s12859-017-1744-3)
Supplement: Supplementary file 1 — LipidMatch Software. The 2017_6_14_LipidMatch_Distribution.zip file contains lipid libraries in .csv format, a batch file for lipidomics with MZmine processing, the LipidMatch R script, and additional helpful R scripts for lipidomics data processing. The .zip file also contains files to guide the user in using LipidMatch, which include: video tutorials, a manual, a trouble shooting document, and example input and output data. For the most up to date version of LipidMatch please visit: http://secim.ufl.edu/secim-tools/. (ZIP 376634 kb) [file 12859_2017_1744_MOESM1_ESM.zip › 2017_6_14_LipidMatch_Distribution/2017_6_15_Trouble_Shooting_Common_Issues.docx]

Trouble Shooting Common Issues

Jeremy Koelmel 6/15/2017

jeremykoelmel@gmail.com

After carefully reading the manual and watching the tutorial videos, please check the following suggestions for LipidMatch errors. If errors continue to persist feel free to contact me for help with your vendor and converted files and feature table (although support is not guaranteed):

**Check Input Files (please see next page for example input directory formats)**

1) Ensure that input files are not contained in a zipped folder

(unzip LipidMatch upon download) and that there is at least one feature table and .ms2 file in the input folder

2) Ensure the following for ddMS^2^ / targeted MS^2^ files:

- Have less than 23 characters in the filename (not including extension)
- Have "dd" somewhere in the filename (not case sensitive)
- End in "n.ms2" or "neg.ms2" for negative mode (not case sensitive)
- End in "p.ms2" or "pos.ms2" for positive mode (not case sensitive)
- Check the file size, if the file is 0 kb there was a problem with the conversion process (see the manual, or an alternative conversion process at the end of this document, and reconvert the file)
- For Agilent files if errors occur after running the code, please try the alternative conversion process (instructions at the end of this document)

3) Check AIF or DIA files (if applicable):

- Have "AIF" somewhere in the filename (not "dd")
- Should end in "n ", "neg ", "p ", or "pos", with a .ms1 or .ms2 extention
- There should be a .ms1 and .ms2 file with the exact same file name

4) For feature tables:

- Make sure that the row identifiers are numeric and unique
- End in "n.csv" or "neg.csv" for negative mode (not case sensitive)
- End in "p.csv" or "pos.csv" for positive mode (not case sensitive)
- When running LipidMatch, be sure to correctly indicate the correct row identifier column, *m/z* column, retention time column, and row that the data starts. Only numeric data should be contained at and after the specified starting data row.

**Running the code:**

- The current version of LipidMatch has been tested on R version 3.3.3. Please download this version of R.
- Make sure to select the entire code before running.
- Pop-up boxes may appear behind other active windows, please minimize all other active windows if you do not see dialogue boxes to select directories and choose parameters.
- Wait: LipidMatch can take minutes to days depending on the computer and number of .ms2 files. Separation of negative/positive files, polarity, and feature tables can make LipidMatch run faster. The STOP sign (at the upper right hand side of the R console) will disappear for longer than 2 minutes when the code is finished, and you should have an output folder with the following files (in the case AIF was not run, and both negative and positive files were included):


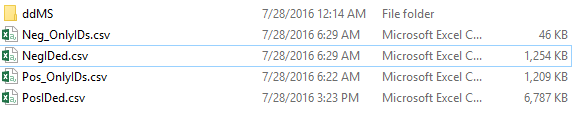


Example of correctly named files in an input folder for LipidMatch:


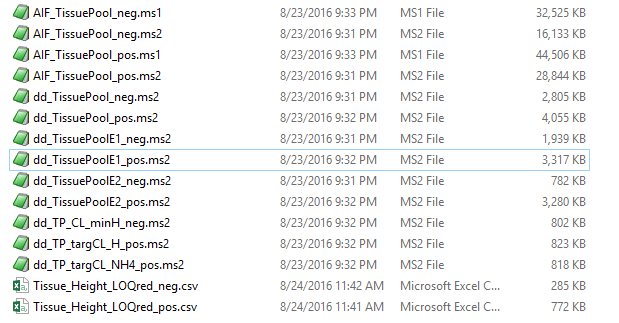


Note that windows often hides file extensions in the file name.

Note that file icons may differ.

Note that no other folders or files should exist within the LipidMatch input folder

A second example of correctly named files in an input folder:


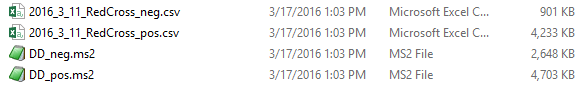


**Non-plasma substrate or acetate system:**

- Currently numerous in-silico libraries for bacterial and non-plasma lipids are contained in LipidMatch. By default, LipidMatch does not search for a number of these lipid classes and does not search for acetate adducts (formate and ammonium adducts are default). If you are running an acetate mobile phase system, you will need to change all formate adducts to FALSE and all acetate adducts to TRUE. Open the LIPID_ID_CRITERIA.csv file within the LipidMatch_Libraries folder. The first column, "CSV File", will show lipid classes, the fourth column, "Run DDA", can be set to TRUE if you wish that lipid to be searched, or FALSE if you do not which to search for that lipid. To aid you can sort by adduct, etc. See tutorial 6 for more information on modifying or adding lipid libraries to LipidMatch.

**Alternative methods for file conversion:**

Try the following method for file conversion, if the one in the written manual is not working:

- Find the "Agilent_mzXML.exe","Agilent_MS2.exe", "Thermo_mzXML.exe", and "Thermo_MS2.exe" files in the subfolder "FileConversion" of your LipidMatch folder. Place these files into your ProteoWizard folder (ProteoWizard can be downloaded from the link in Step 1). Make sure when you double click the .exe files that you choose the following parameters as shown in the screen captures below and then click Save & Exit. You can add parameters by clicking the filter icon on the top right of ms2mz. The order of the parameters does matter, and should be the same as in the screen shots. Do not change the .exe name or else the parameters will be erased.

Agilent_MS2.exe
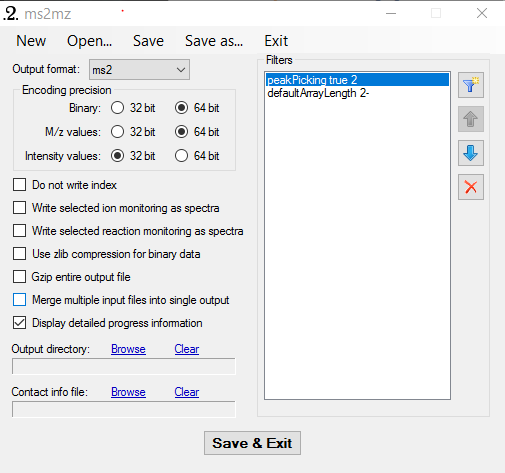


Agilent_mzXML.exe
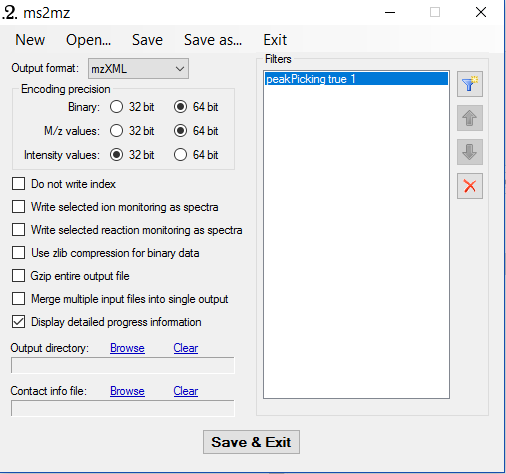


Thermo_MS2.exe
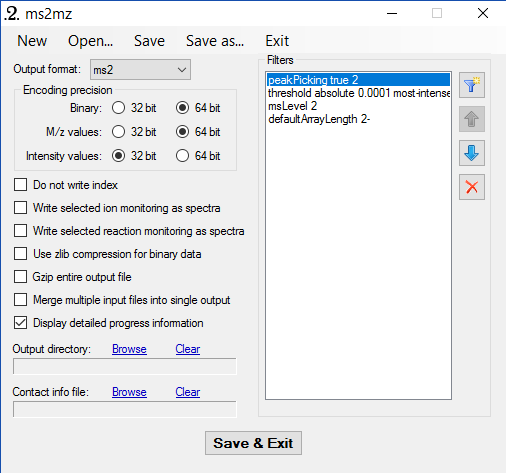


Thermo_mzXML.exe
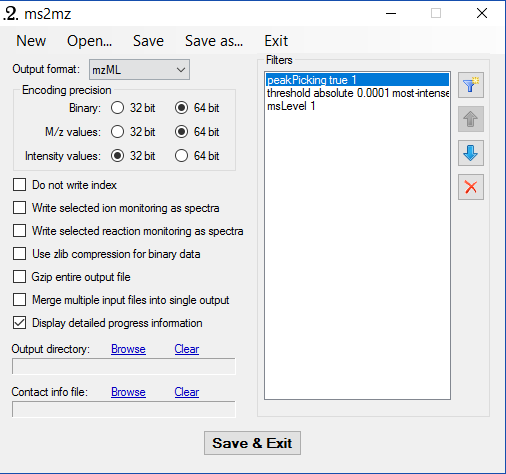


- Drag Agilent files (.d) or Thermo (.raw) files onto the correct .exe file. The converted .ms2 and .mzXML files will appear in their original directory. The .mzXML files are used for peak picking in MZmine (if you use alternative peak picking software you do not need .mzXML files). The .ms2 files are used by LipidMatch. Hence vendor files which contain MS/MS data for lipid annotation should be converted into .ms2 format.
