# Supplementary figures and images for: LipidMatch: an automated workflow for rule-based lipid identification using untargeted high-resolution tandem mass spectrometry data
Source: BMC Bioinformatics. 2017 Jul 10;18:331. doi: 10.1186/s12859-017-1744-3 (PMC5504796; doi:10.1186/s12859-017-1744-3)

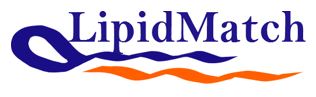

Supplement: Supplementary file 1 — LipidMatch Software. The 2017_6_14_LipidMatch_Distribution.zip file contains lipid libraries in .csv format, a batch file for lipidomics with MZmine processing, the LipidMatch R script, and additional helpful R scripts for lipidomics data processing. The .zip file also contains files to guide the user in using LipidMatch, which include: video tutorials, a manual, a trouble shooting document, and example input and output data. For the most up to date version of LipidMatch please visit: http://secim.ufl.edu/secim-tools/. (ZIP 376634 kb) [file 12859_2017_1744_MOESM1_ESM.zip › 2017_6_14_LipidMatch_Distribution/Additional_Tools/LipidMatch_Icon/LipidMatch_Icon.JPG]

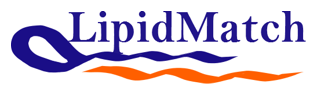

Supplement: Supplementary file 1 — LipidMatch Software. The 2017_6_14_LipidMatch_Distribution.zip file contains lipid libraries in .csv format, a batch file for lipidomics with MZmine processing, the LipidMatch R script, and additional helpful R scripts for lipidomics data processing. The .zip file also contains files to guide the user in using LipidMatch, which include: video tutorials, a manual, a trouble shooting document, and example input and output data. For the most up to date version of LipidMatch please visit: http://secim.ufl.edu/secim-tools/. (ZIP 376634 kb) [file 12859_2017_1744_MOESM1_ESM.zip › 2017_6_14_LipidMatch_Distribution/Additional_Tools/LipidMatch_Icon/LipidMatch_Icon.PNG]

## Slide 1
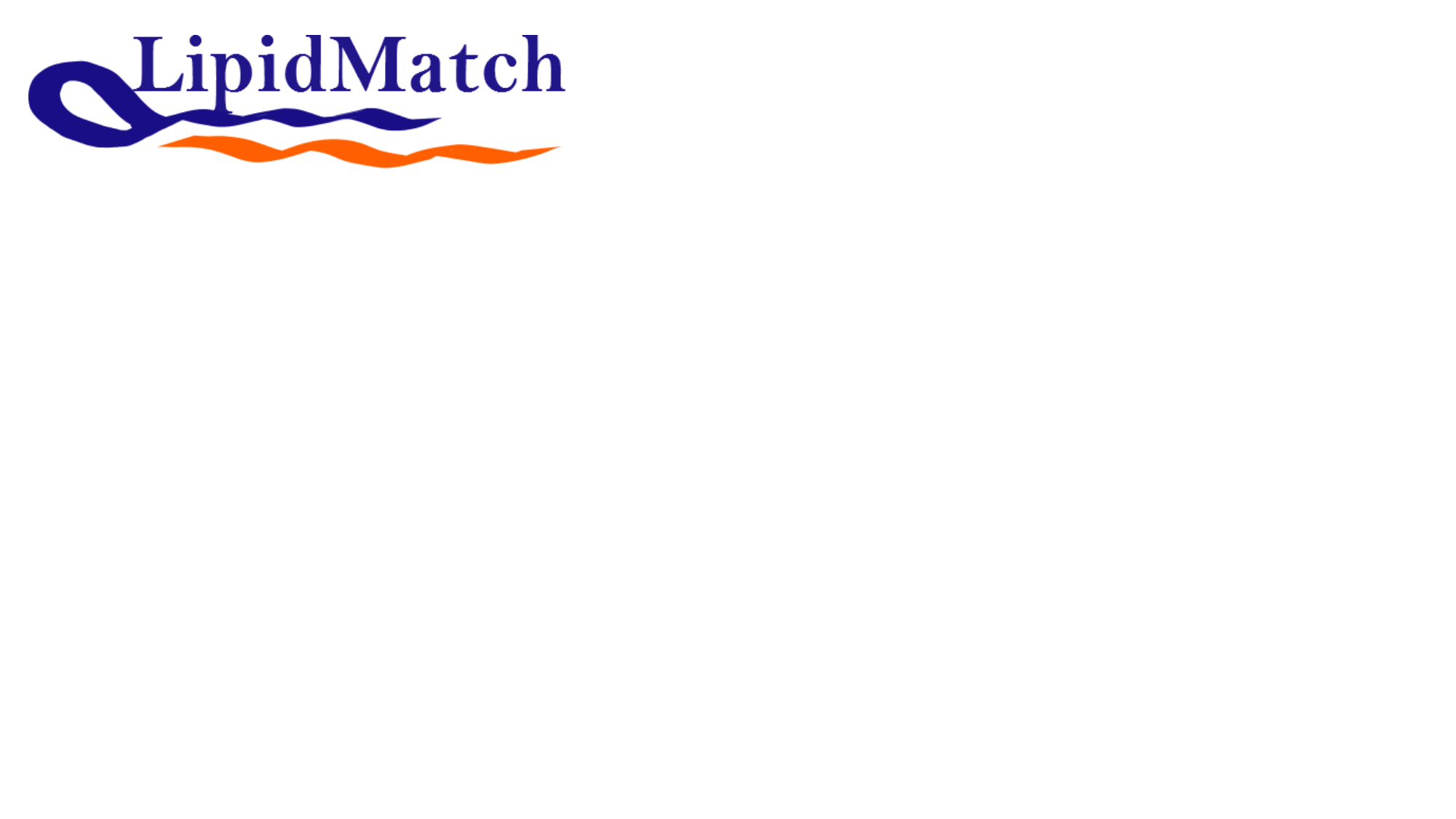

Supplement: Supplementary file 1 — LipidMatch Software. The 2017_6_14_LipidMatch_Distribution.zip file contains lipid libraries in .csv format, a batch file for lipidomics with MZmine processing, the LipidMatch R script, and additional helpful R scripts for lipidomics data processing. The .zip file also contains files to guide the user in using LipidMatch, which include: video tutorials, a manual, a trouble shooting document, and example input and output data. For the most up to date version of LipidMatch please visit: http://secim.ufl.edu/secim-tools/. (ZIP 376634 kb) [file 12859_2017_1744_MOESM1_ESM.zip › 2017_6_14_LipidMatch_Distribution/Additional_Tools/LipidMatch_Icon/LipidMatch_Icon.pptx]
