# Supplementary material for: LipidMatch: an automated workflow for rule-based lipid identification using untargeted high-resolution tandem mass spectrometry data
Source: BMC Bioinformatics. 2017 Jul 10;18:331. doi: 10.1186/s12859-017-1744-3 (PMC5504796; doi:10.1186/s12859-017-1744-3)
Supplement: Supplementary file 2 — Supplemental Figures and Tables. Contains Figure S1 through Figure S5, and Table S1 through Table S3. (PPTX 593 kb) [file 12859_2017_1744_MOESM2_ESM.pptx]

## Slide 1
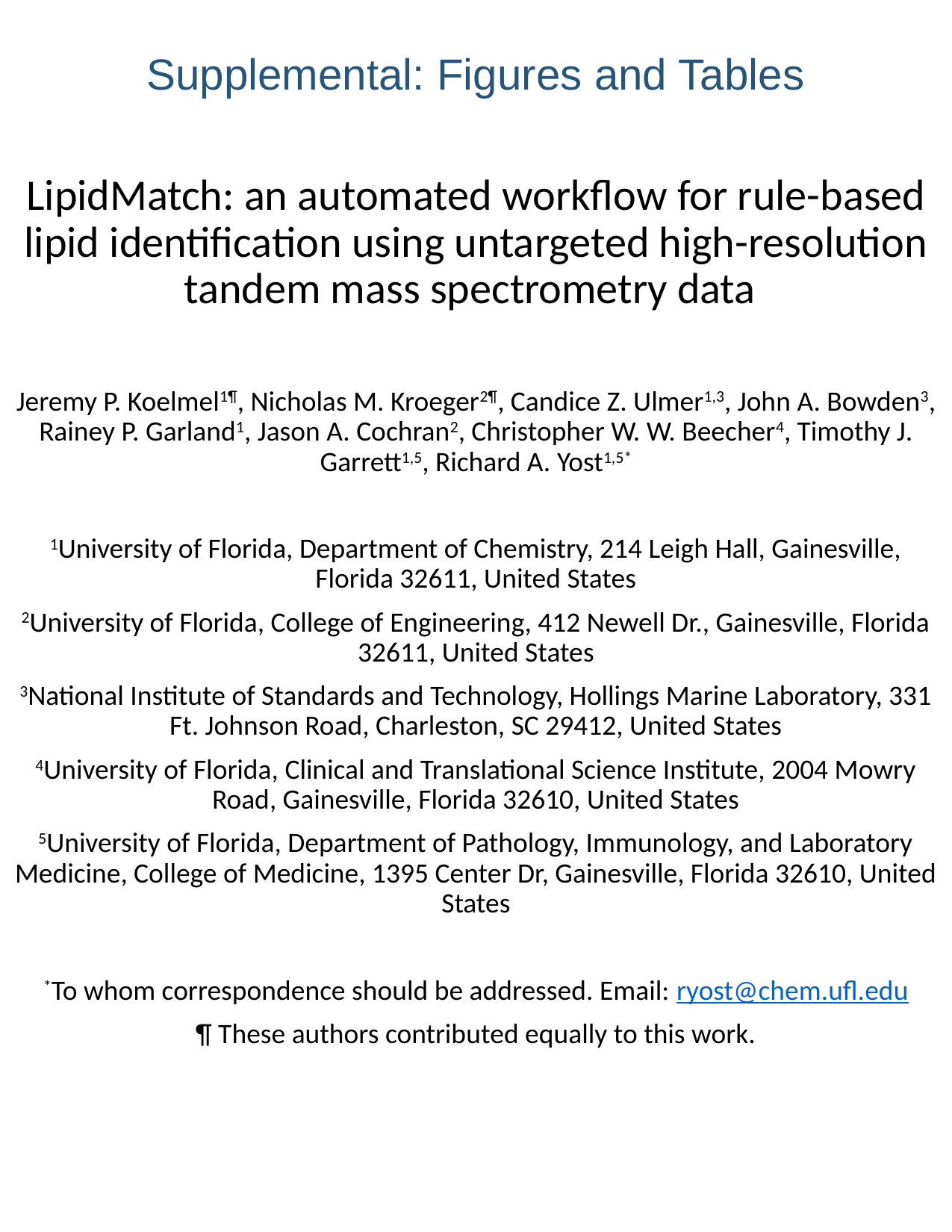

Supplemental: Figures and Tables
LipidMatch: an automated workflow for rule-based lipid identification using untargeted high-resolution tandem mass spectrometry data
Jeremy P. Koelmel1¶, Nicholas M. Kroeger2¶, Candice Z. Ulmer1,3, John A. Bowden3, Rainey P. Garland1, Jason A. Cochran2, Christopher W. W. Beecher4, Timothy J. Garrett1,5, Richard A. Yost1,5*
1University of Florida, Department of Chemistry, 214 Leigh Hall, Gainesville, Florida 32611, United States
2University of Florida, College of Engineering, 412 Newell Dr., Gainesville, Florida 32611, United States
3National Institute of Standards and Technology, Hollings Marine Laboratory, 331 Ft. Johnson Road, Charleston, SC 29412, United States
4University of Florida, Clinical and Translational Science Institute, 2004 Mowry Road, Gainesville, Florida 32610, United States
5University of Florida, Department of Pathology, Immunology, and Laboratory Medicine, College of Medicine, 1395 Center Dr, Gainesville, Florida 32610, United States
*To whom correspondence should be addressed. Email: ryost@chem.ufl.edu
¶ These authors contributed equally to this work.

## Slide 2
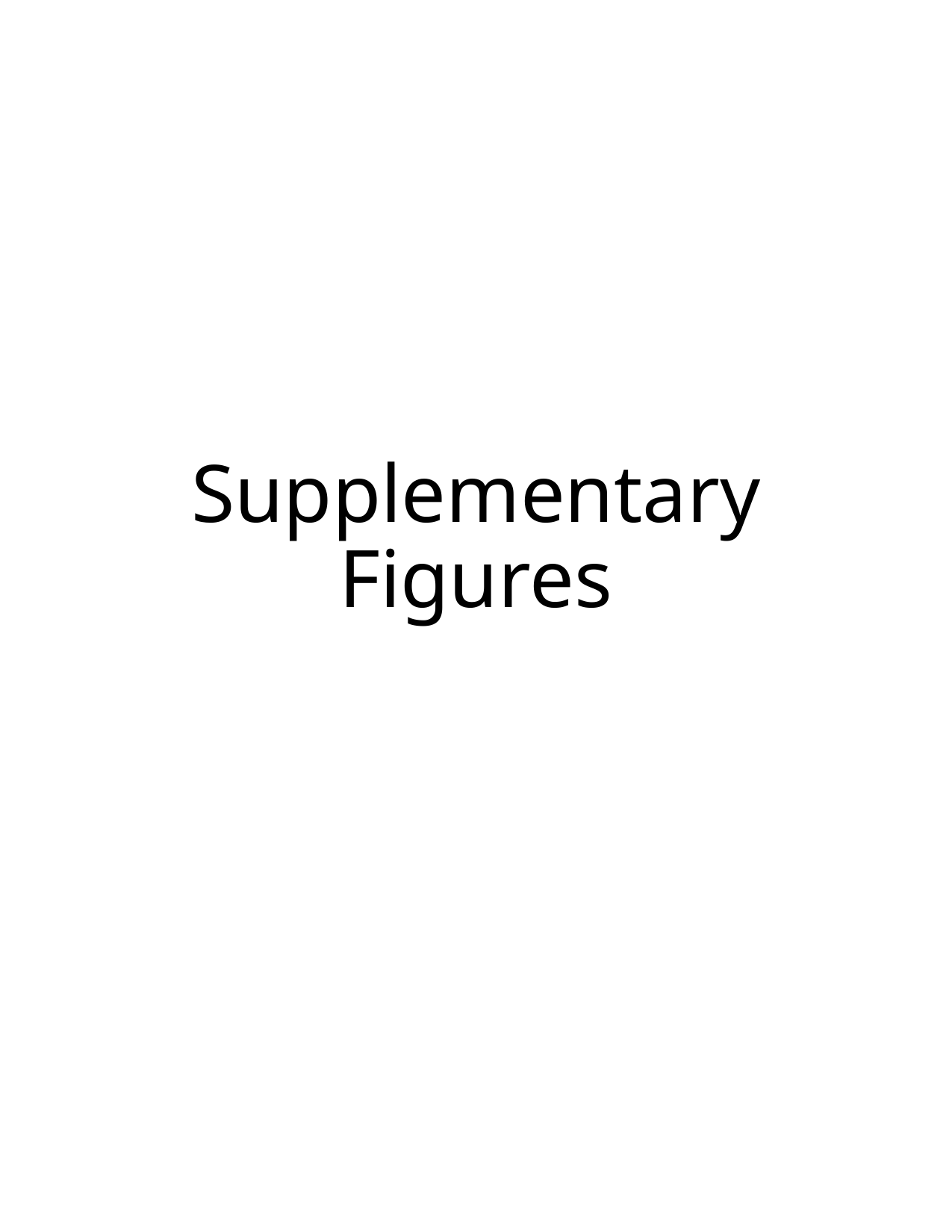

# Supplementary Figures

## Slide 3
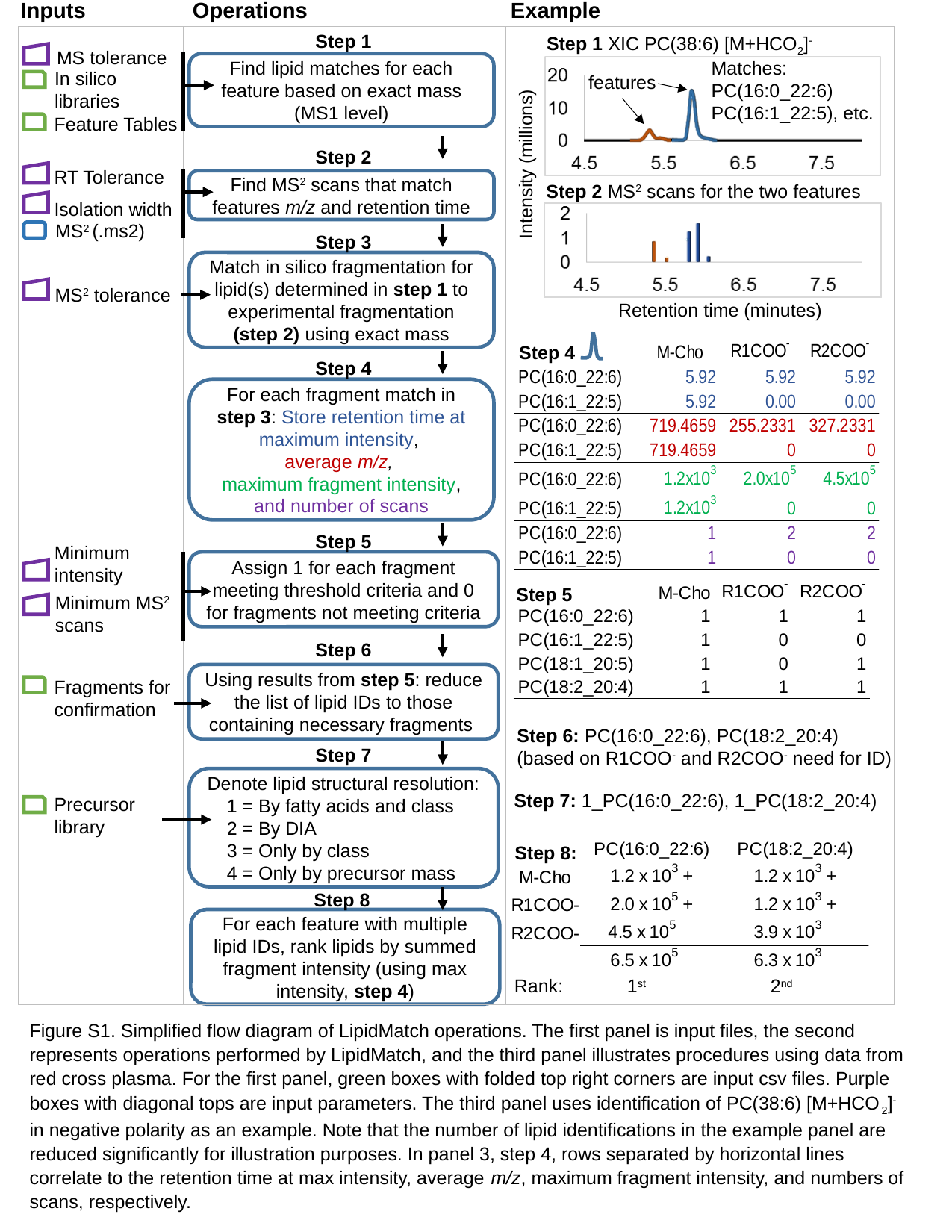

Inputs
Operations
Example
Step 1
MS tolerance
Find lipid matches for each feature based on exact mass (MS1 level)
In silico
libraries
Feature Tables
Step 1 XIC PC(38:6) [M+HCO2]-
Matches:
PC(16:0_22:6)
PC(16:1_22:5), etc.
features
Step 2
RT Tolerance
Find MS2 scans that match features m/z and retention time
Isolation width
MS2 (.ms2)
Intensity (millions)
Step 2 MS2 scans for the two features
Step 3
Match in silico fragmentation for lipid(s) determined in step 1 to experimental fragmentation (step 2) using exact mass
MS2 tolerance
Retention time (minutes)
Step 4
Step 4
For each fragment match in step 3: Store retention time at maximum intensity,
average m/z,
maximum fragment intensity, and number of scans
Step 5
Minimum
intensity
Assign 1 for each fragment meeting threshold criteria and 0 for fragments not meeting criteria
Minimum MS2
scans
Step 5
Step 6
Using results from step 5: reduce the list of lipid IDs to those containing necessary fragments
Fragments for
confirmation
Step 6: PC(16:0_22:6), PC(18:2_20:4)
(based on R1COO- and R2COO- need for ID)
Step 7
Denote lipid structural resolution:
 1 = By fatty acids and class
 2 = By DIA
 3 = Only by class
 4 = Only by precursor mass
Precursor
library
Step 7: 1_PC(16:0_22:6), 1_PC(18:2_20:4)
Step 8:
Step 8
For each feature with multiple lipid IDs, rank lipids by summed fragment intensity (using max intensity, step 4)
2nd
Rank:
1st
Figure S1. Simplified flow diagram of LipidMatch operations. The first panel is input files, the second represents operations performed by LipidMatch, and the third panel illustrates procedures using data from red cross plasma. For the first panel, green boxes with folded top right corners are input csv files. Purple boxes with diagonal tops are input parameters. The third panel uses identification of PC(38:6) [M+HCO2]- in negative polarity as an example. Note that the number of lipid identifications in the example panel are reduced significantly for illustration purposes. In panel 3, step 4, rows separated by horizontal lines correlate to the retention time at max intensity, average m/z, maximum fragment intensity, and numbers of scans, respectively.

## Slide 4
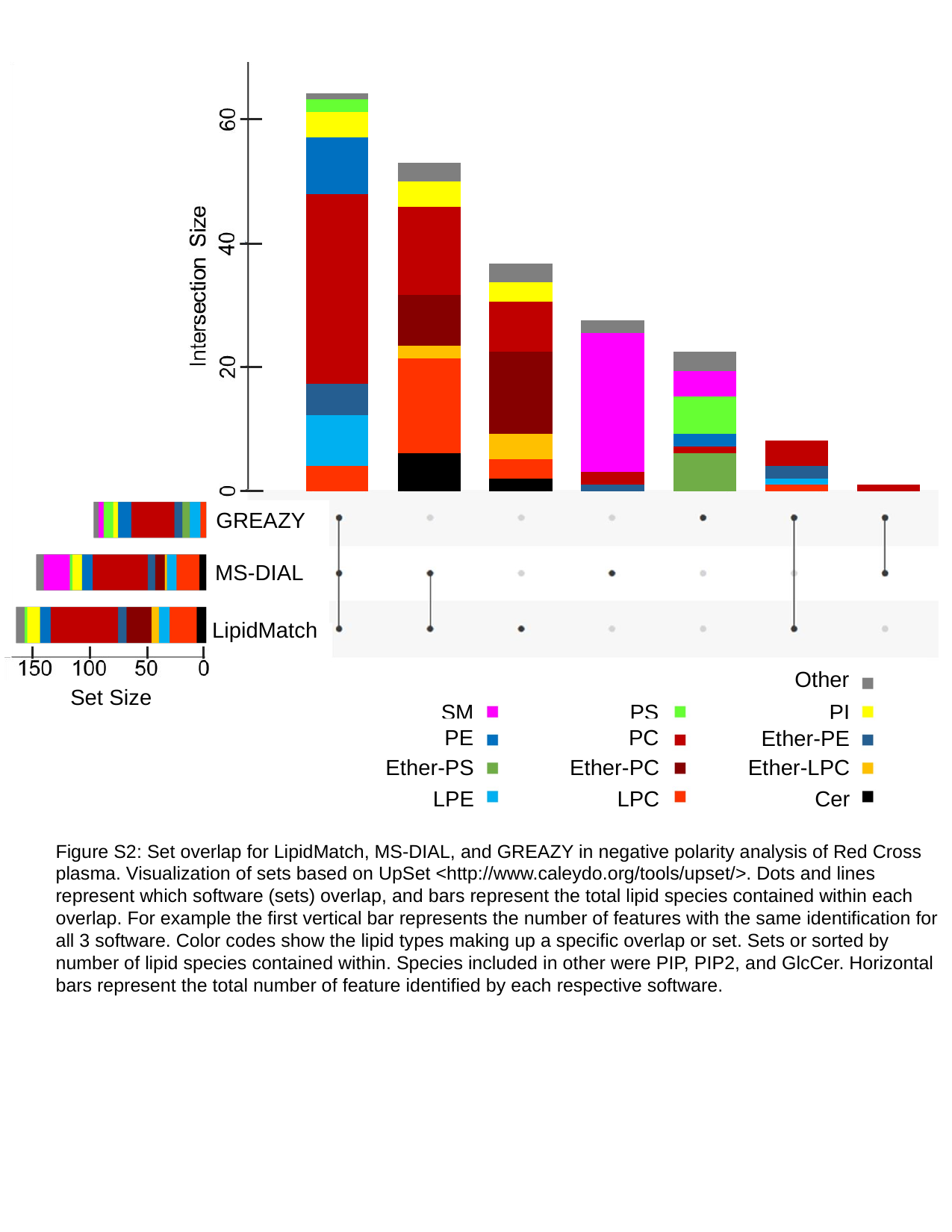

GREAZY
MS-DIAL
LipidMatch
Other
Set Size
SM
PS
PI
PE
PC
Ether-PE
Ether-PS
Ether-PC
Ether-LPC
LPE
LPC
Cer
Figure S2: Set overlap for LipidMatch, MS-DIAL, and GREAZY in negative polarity analysis of Red Cross plasma. Visualization of sets based on UpSet <http://www.caleydo.org/tools/upset/>. Dots and lines represent which software (sets) overlap, and bars represent the total lipid species contained within each overlap. For example the first vertical bar represents the number of features with the same identification for all 3 software. Color codes show the lipid types making up a specific overlap or set. Sets or sorted by number of lipid species contained within. Species included in other were PIP, PIP2, and GlcCer. Horizontal bars represent the total number of feature identified by each respective software.

## Slide 5
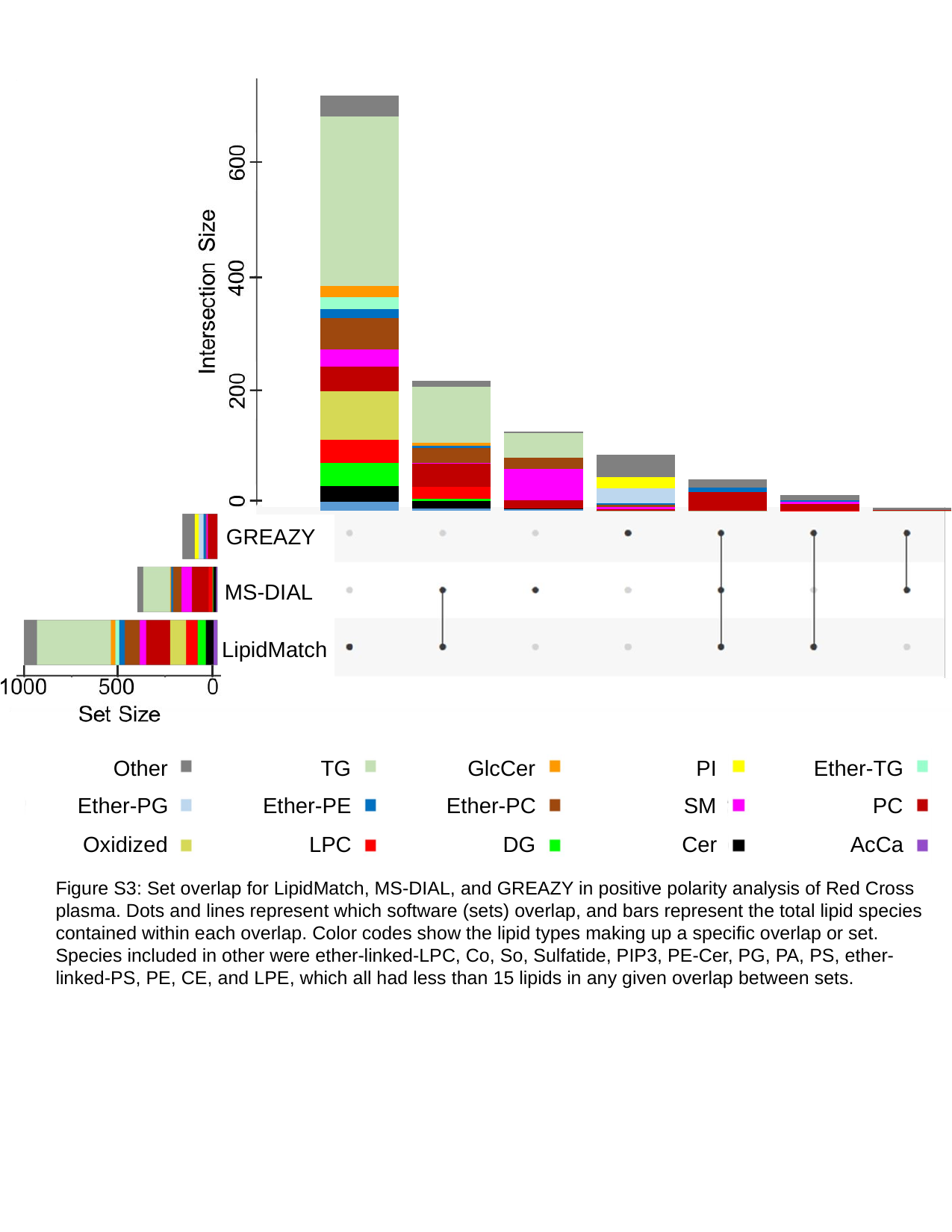

### Chart
| Category | AcCa | Cer | DG | LPC | Oxidized | PC | SM | ether-linked-PC | ether-linked-PE | ether-linked-PG | ether-linked-TG | PI | GlcCer | TG | other |
|---|---|---|---|---|---|---|---|---|---|---|---|---|---|---|---|
| Only LipidMatch | 16.0 | 26.0 | 38.0 | 39.0 | 82.0 | 41.0 | 29.0 | 52.0 | 16.0 | 0.0 | 20.0 | 0.0 | 18.0 | 285.0 | 35.0 |
| LipidMatch & MS-DIAL | 4.0 | 13.0 | 4.0 | 20.0 | 0.0 | 38.0 | 2.0 | 25.0 | 3.0 | 0.0 | 0.0 | 0.0 | 5.0 | 95.0 | 10.0 |
| Only MSDIAL | 3.0 | 1.0 | 0.0 | 0.0 | 0.0 | 14.0 | 52.0 | 19.0 | 0.0 | 0.0 | 0.0 | 0.0 | 0.0 | 42.0 | 2.0 |
| Only GREAZY | 0.0 | 0.0 | 0.0 | 0.0 | 0.0 | 3.0 | 4.0 | 3.0 | 3.0 | 25.0 | 0.0 | 19.0 | 0.0 | 0.0 | 38.0 |
| All | 0.0 | 0.0 | 0.0 | 0.0 | 0.0 | 32.0 | 0.0 | 0.0 | 7.0 | 0.0 | 0.0 | 0.0 | 0.0 | 0.0 | 14.0 |
| LipidMatch & GREAZY | 0.0 | 0.0 | 0.0 | 1.0 | 0.0 | 11.0 | 3.0 | 0.0 | 3.0 | 0.0 | 0.0 | 0.0 | 0.0 | 0.0 | 9.0 |
| MS-DIAL & GREAZY | 0.0 | 0.0 | 0.0 | 0.0 | 0.0 | 2.0 | 0.0 | 0.0 | 0.0 | 0.0 | 0.0 | 0.0 | 0.0 | 0.0 | 3.0 |
GREAZY
MS-DIAL
LipidMatch
Other
TG
GlcCer
PI
Ether-TG
Ether-PG
Ether-PE
Ether-PC
SM
PC
Oxidized
LPC
DG
Cer
AcCa
Figure S3: Set overlap for LipidMatch, MS-DIAL, and GREAZY in positive polarity analysis of Red Cross plasma. Dots and lines represent which software (sets) overlap, and bars represent the total lipid species contained within each overlap. Color codes show the lipid types making up a specific overlap or set. Species included in other were ether-linked-LPC, Co, So, Sulfatide, PIP3, PE-Cer, PG, PA, PS, ether-linked-PS, PE, CE, and LPE, which all had less than 15 lipids in any given overlap between sets.

## Slide 6
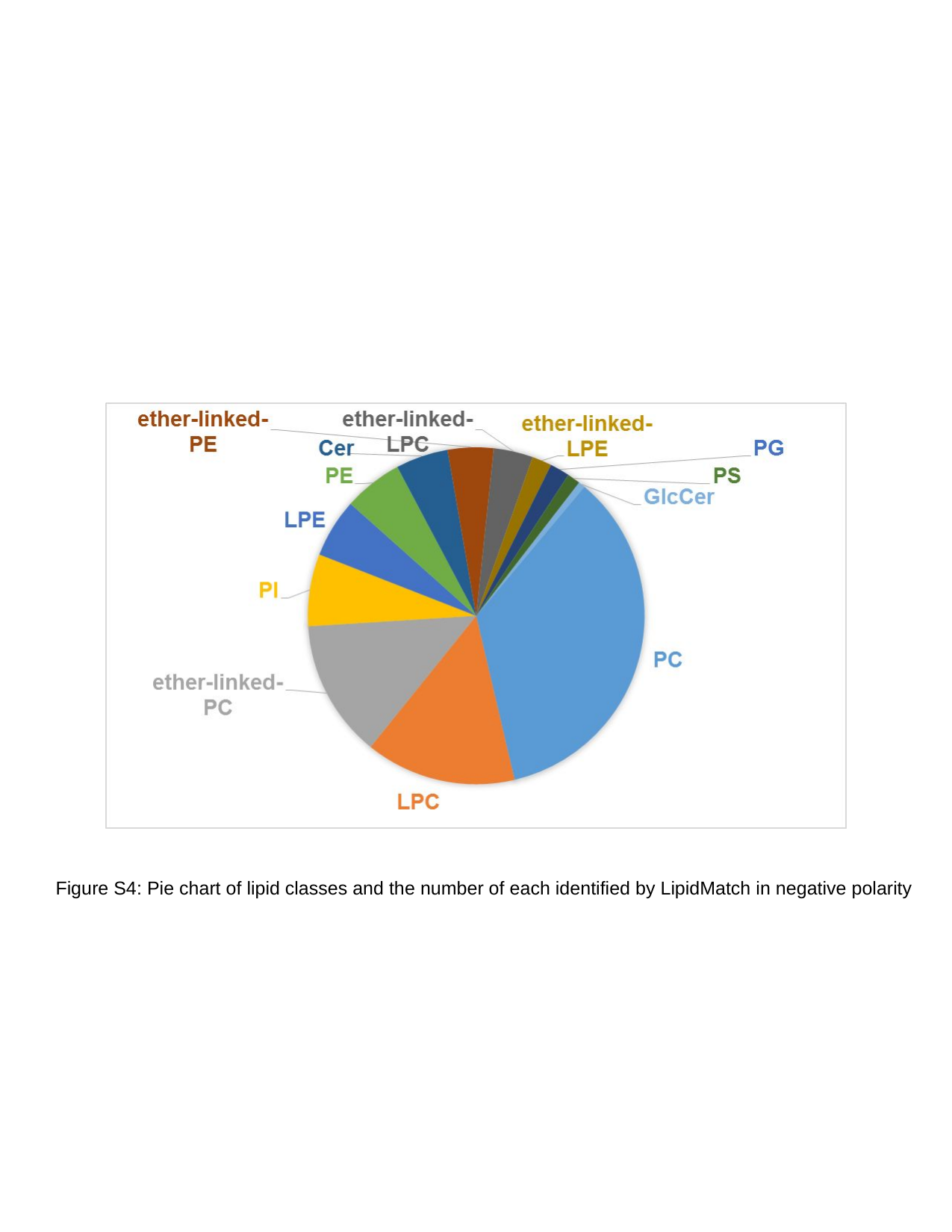

Figure S4: Pie chart of lipid classes and the number of each identified by LipidMatch in negative polarity

## Slide 7
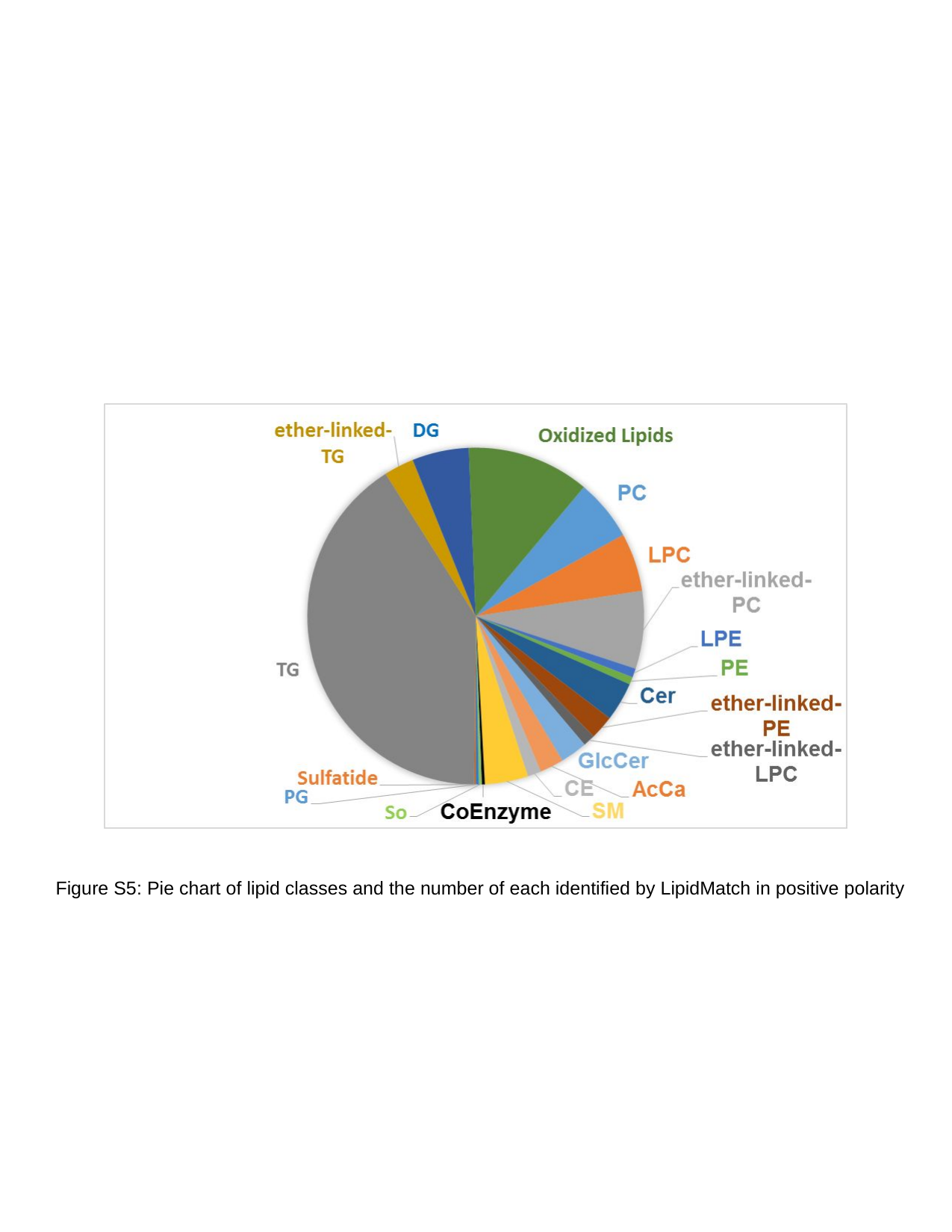

Figure S5: Pie chart of lipid classes and the number of each identified by LipidMatch in positive polarity

## Slide 8
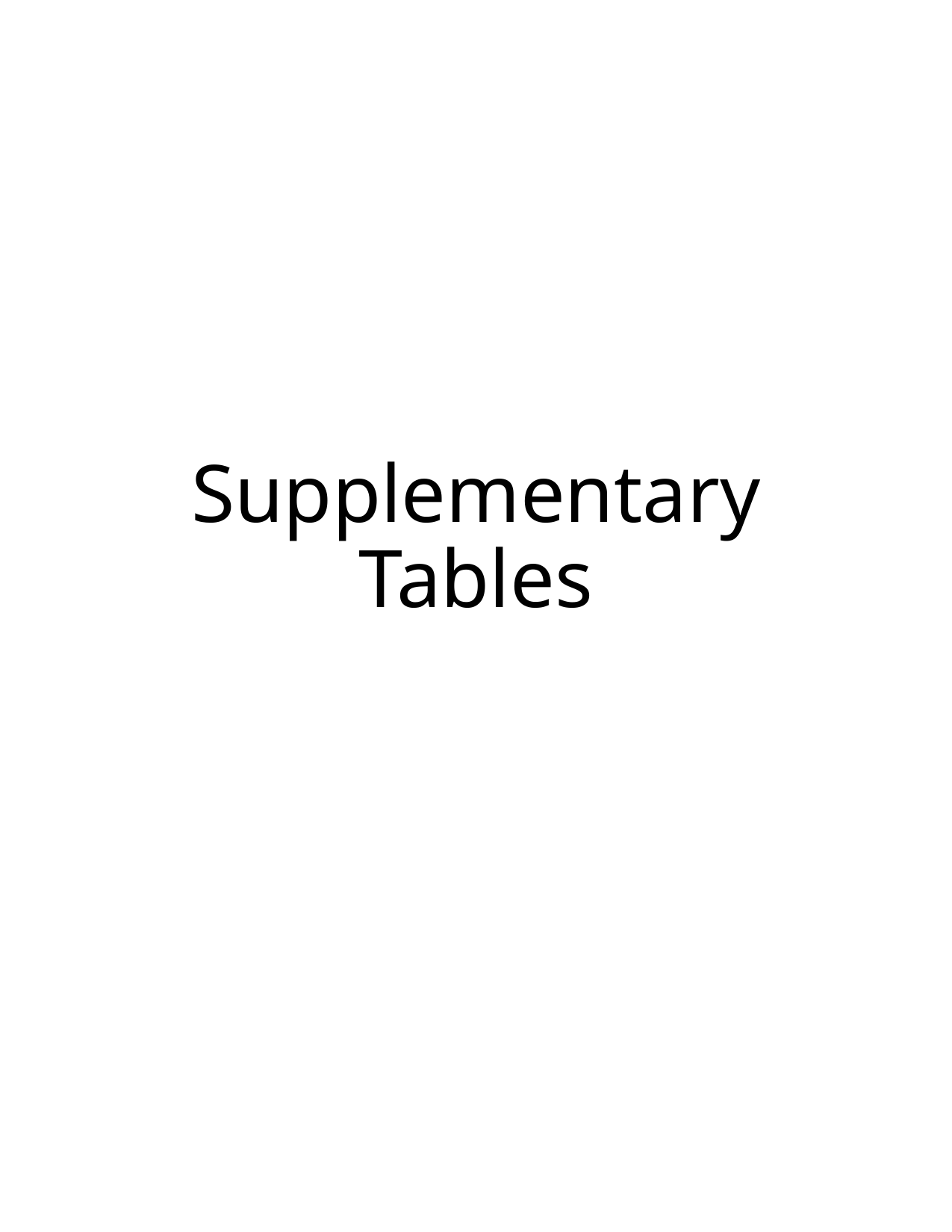

# Supplementary Tables

## Slide 9
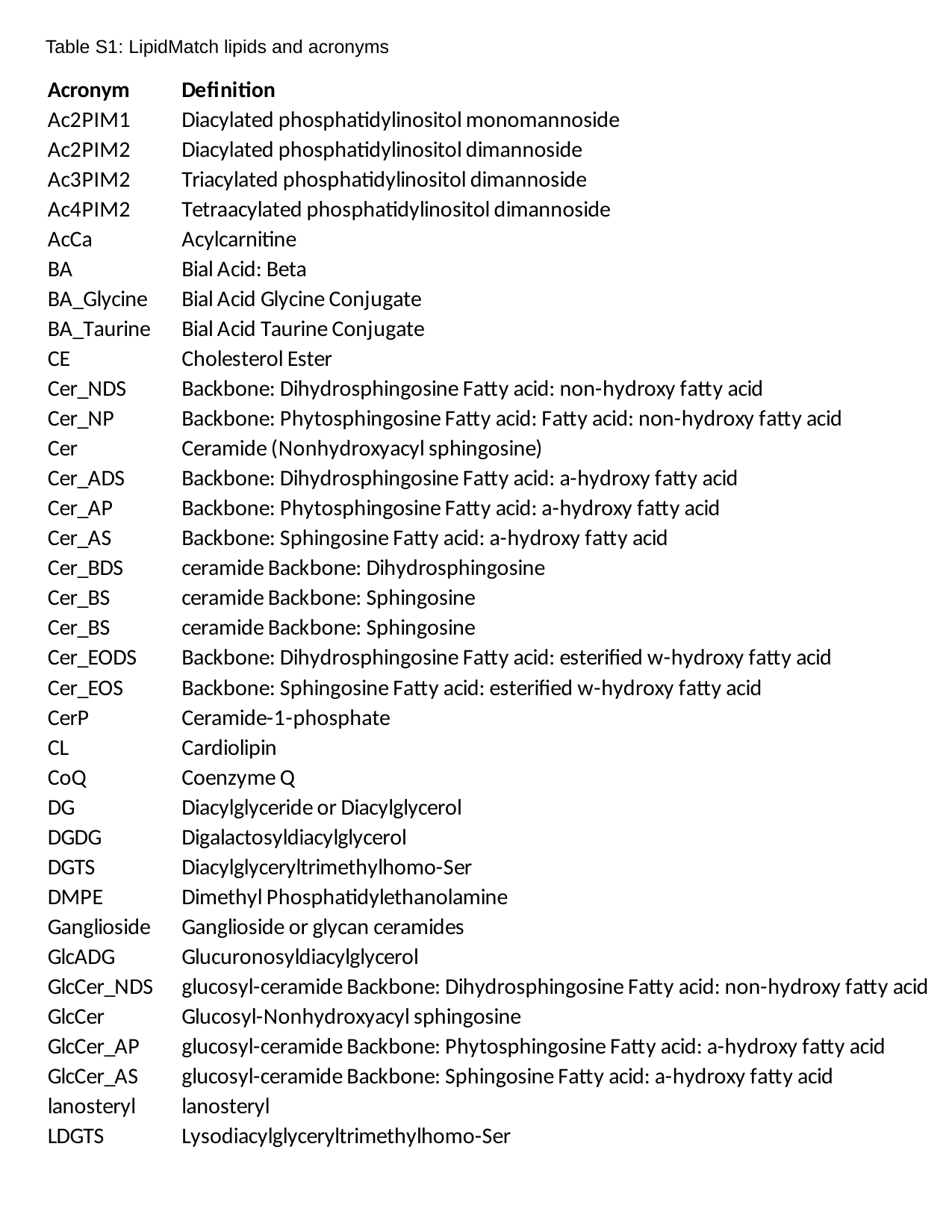

Table S1: LipidMatch lipids and acronyms

## Slide 10
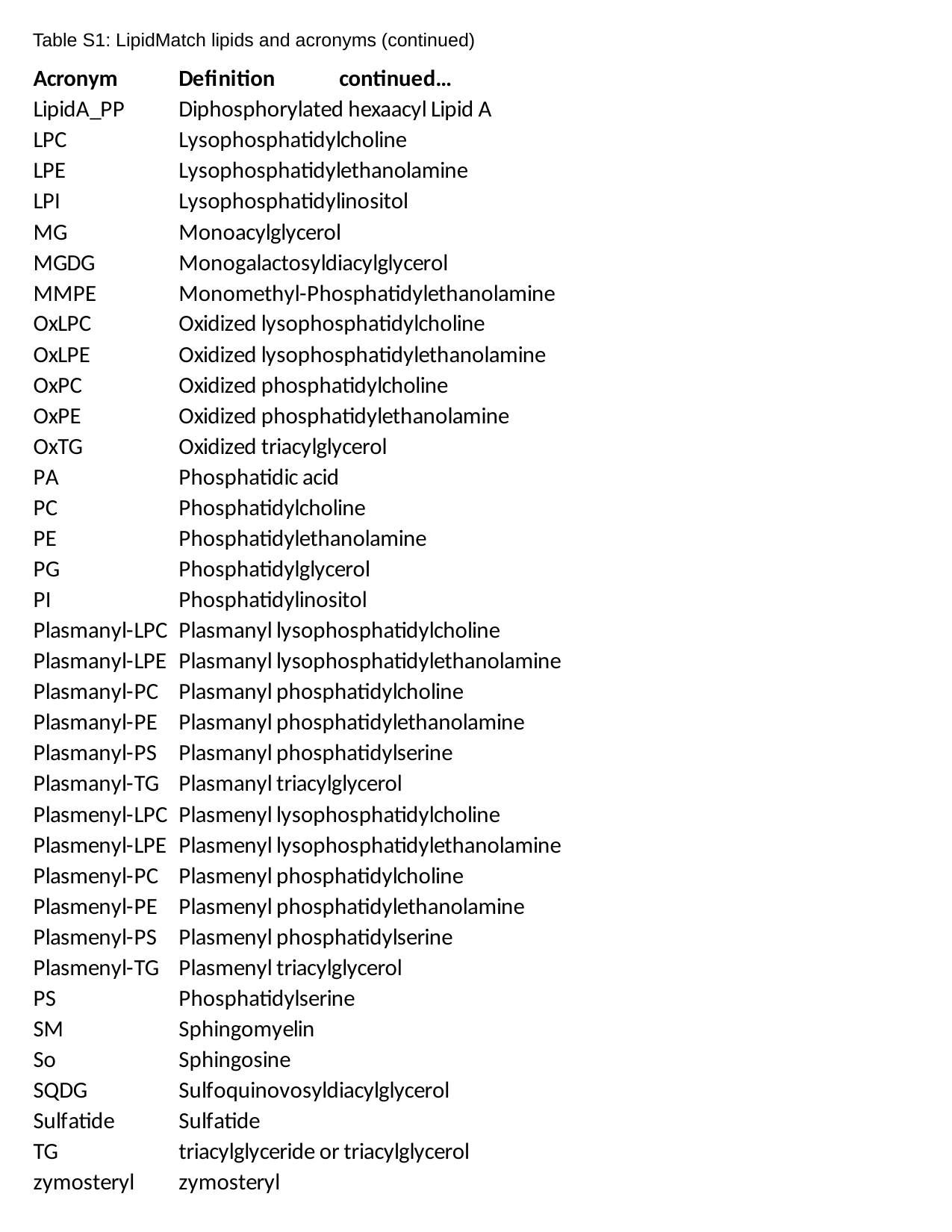

Table S1: LipidMatch lipids and acronyms (continued)

## Slide 11
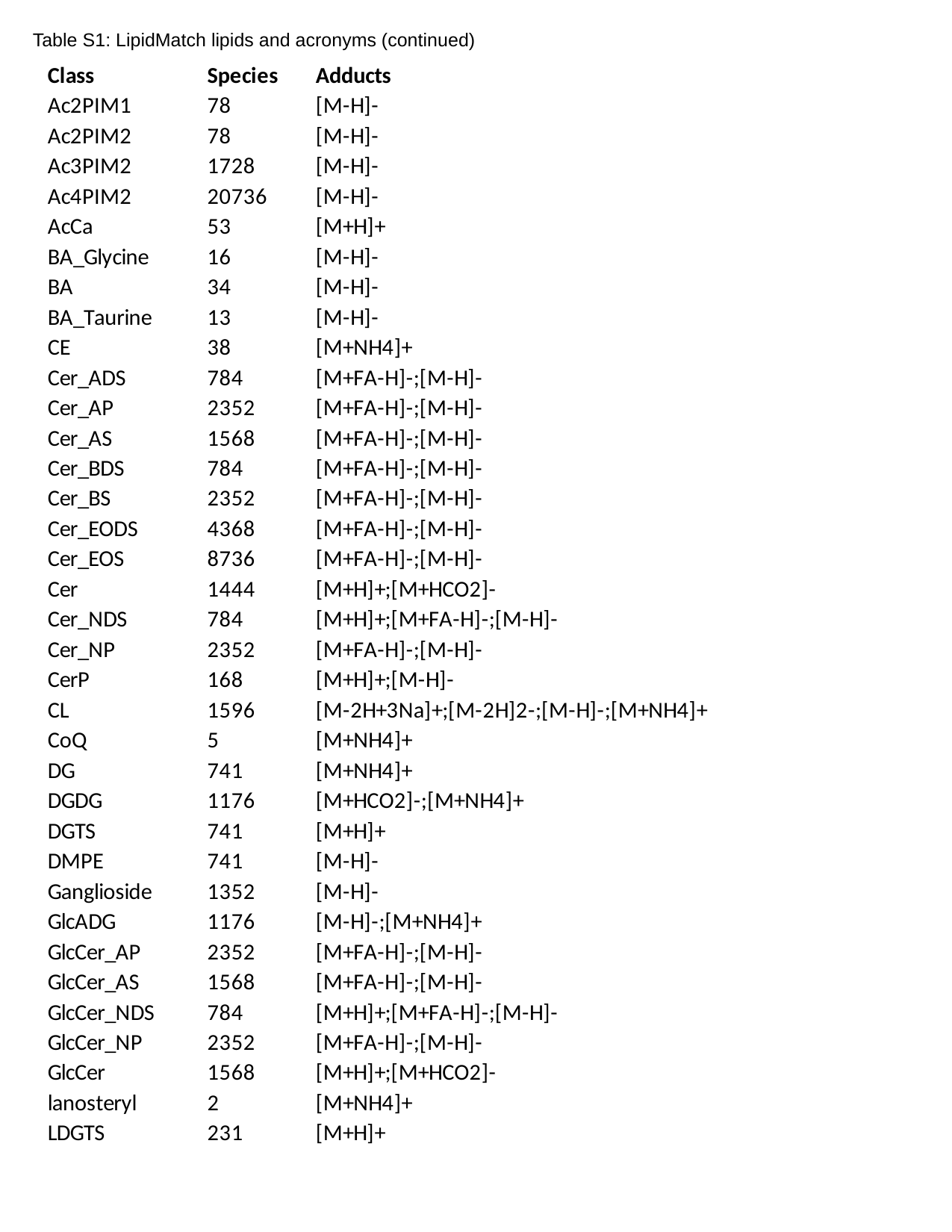

Table S1: LipidMatch lipids and acronyms (continued)

## Slide 12
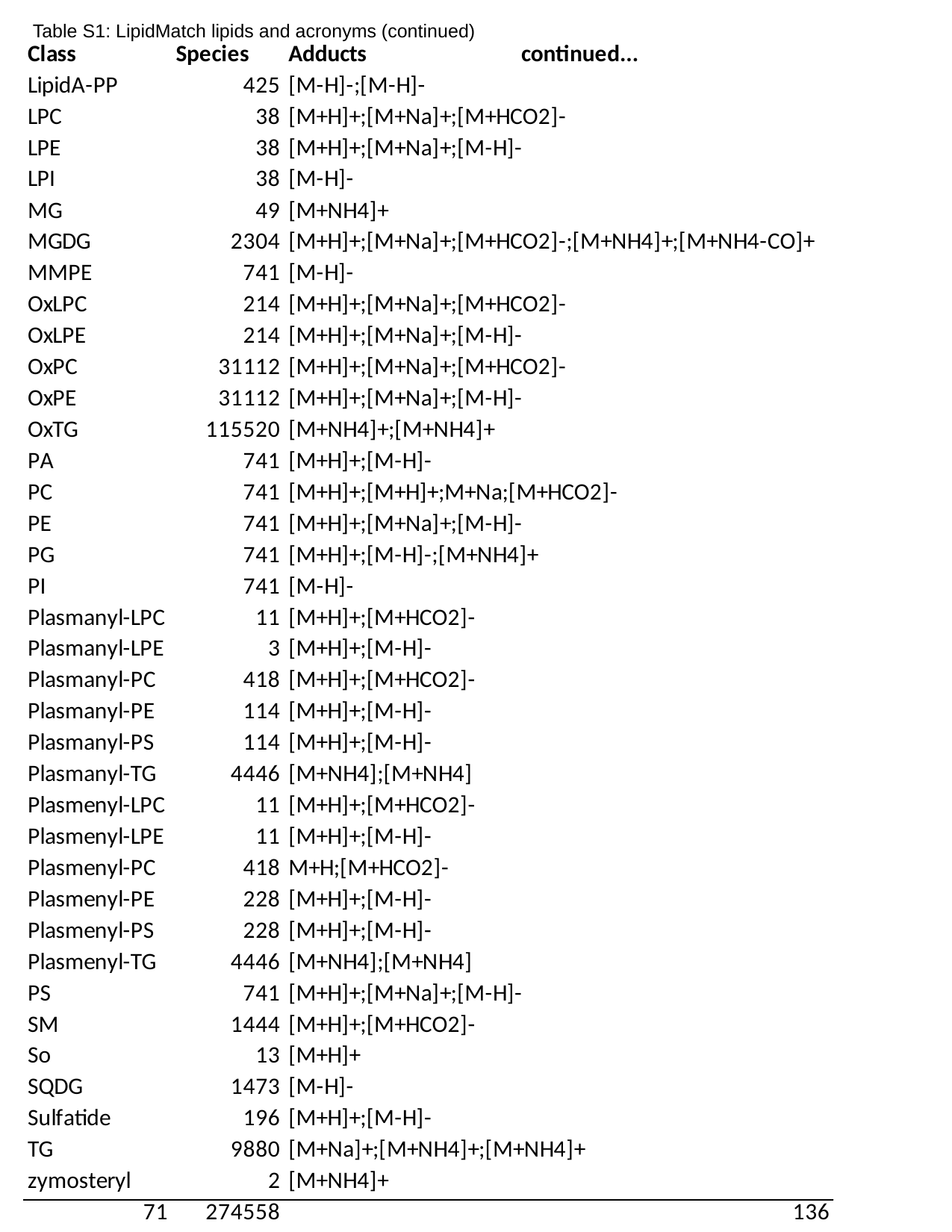

Table S1: LipidMatch lipids and acronyms (continued)

## Slide 13
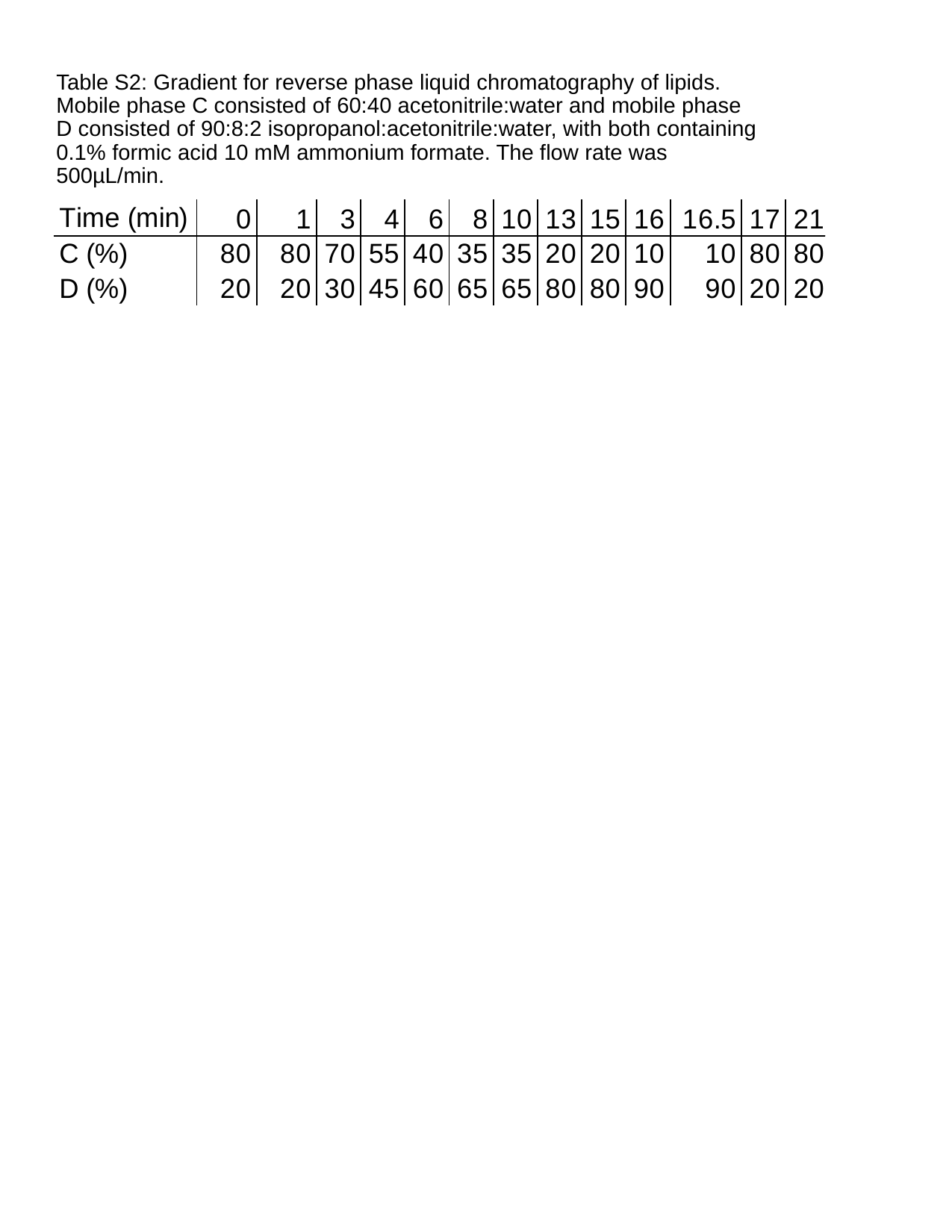

Table S2: Gradient for reverse phase liquid chromatography of lipids. Mobile phase C consisted of 60:40 acetonitrile:water and mobile phase D consisted of 90:8:2 isopropanol:acetonitrile:water, with both containing 0.1% formic acid 10 mM ammonium formate. The flow rate was 500µL/min.

## Slide 14
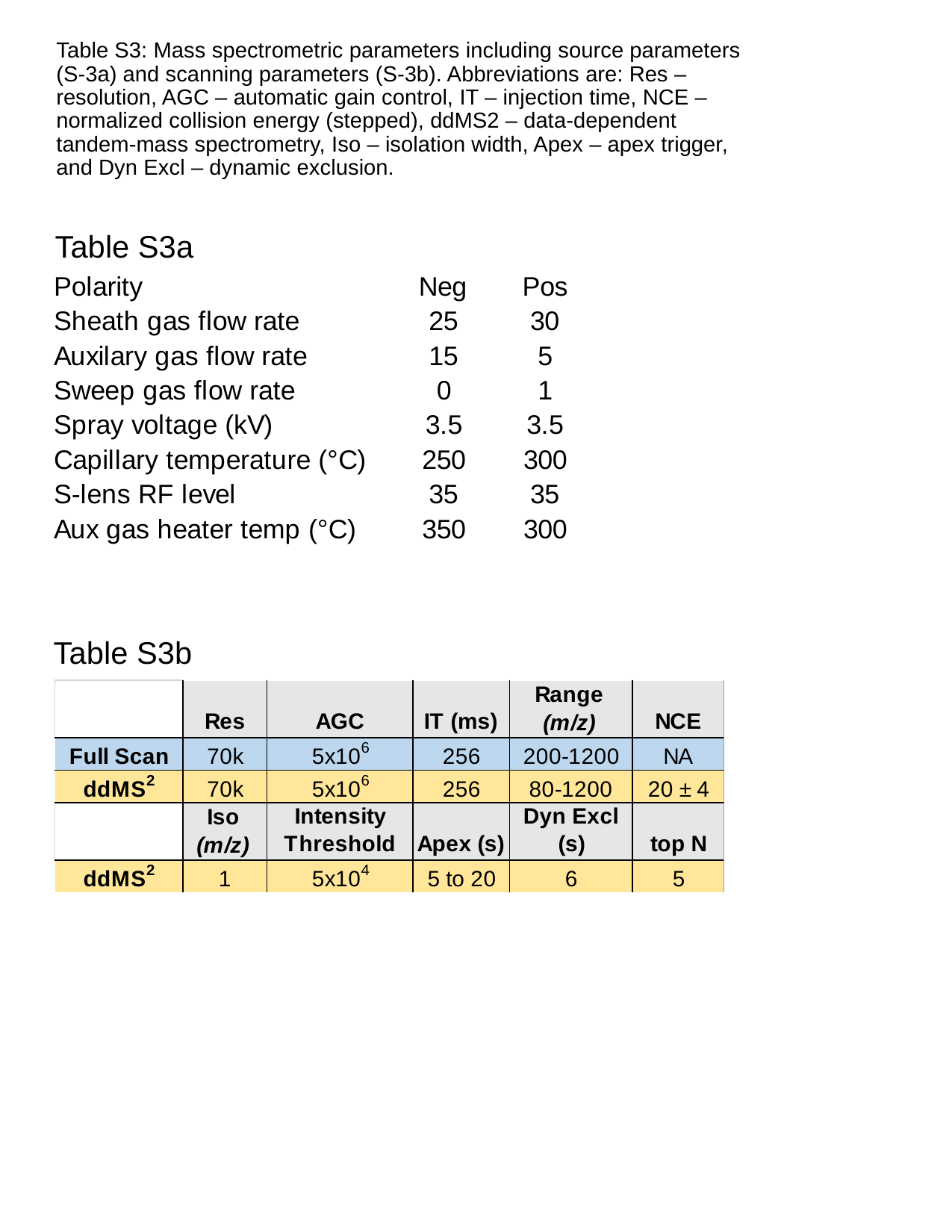

Table S3: Mass spectrometric parameters including source parameters (S-3a) and scanning parameters (S-3b). Abbreviations are: Res – resolution, AGC – automatic gain control, IT – injection time, NCE – normalized collision energy (stepped), ddMS2 – data-dependent tandem-mass spectrometry, Iso – isolation width, Apex – apex trigger, and Dyn Excl – dynamic exclusion.
Table S3a
Table S3b
