# Supplementary material for: LipidMatch: an automated workflow for rule-based lipid identification using untargeted high-resolution tandem mass spectrometry data
Source: BMC Bioinformatics. 2017 Jul 10;18:331. doi: 10.1186/s12859-017-1744-3 (PMC5504796; doi:10.1186/s12859-017-1744-3)
Supplement: Supplementary file 1 — LipidMatch Software. The 2017_6_14_LipidMatch_Distribution.zip file contains lipid libraries in .csv format, a batch file for lipidomics with MZmine processing, the LipidMatch R script, and additional helpful R scripts for lipidomics data processing. The .zip file also contains files to guide the user in using LipidMatch, which include: video tutorials, a manual, a trouble shooting document, and example input and output data. For the most up to date version of LipidMatch please visit: http://secim.ufl.edu/secim-tools/. (ZIP 376634 kb) [file 12859_2017_1744_MOESM1_ESM.zip › 2017_6_14_LipidMatch_Distribution/Tutorial_Videos/Read_Me_Before_Watching.docx]

LipidMatch Video Tutorials

Jeremy P. Koelmel, Nicholas M. Kroeger, and Candice Z. Ulmer, Ph.D.

The video tutorials in this folder are in-depth and will guide you step by step through all processes needed to run LipidMatch. Note that the videos are updated less frequently than the user manual ([date]LipidMatch_Manual.docx), and hence please refer to the .docx user manual for updated user instructions.

The following updates are not reflected in the videos:

- R version 3.3.3 should be used, not the latest version as suggested in the video tutorial.
- The video tutorial focuses on a workflow using Thermo files, although most steps are the same for any vendor format.
  - For Agilent files please see the [date]Trouble_Shooting_Common_Issues.docx for instructions on converting the .d files. Use 1 for minimum fragment intensity for Agilent files.
